# Activity of silver-zinc nanozeolite-based antibiofilm wound dressings in an in vitro biofilm model and comparison with commercial dressings

**DOI:** 10.1186/s11671-025-04208-8

**Published:** 2025-02-11

**Authors:** Sarah Abdulaziz Alobaid, Sweta Shrestha, Morgan Tasseff, Bo Wang, Monique L. van Hoek, Prabir K. Dutta

**Affiliations:** 1 School of Systems Biology, George Mason University, Manassas, VA 20110 USA; 2 Zeovation Inc., Columbus, OH 43212 USA; 3 Department of Chemistry and Biochemistry, The Ohio State University, Columbus, OH 43210 USA

**Keywords:** Chronic wounds, Quaternary ammonium, Quat, Silver, Zinc, Antimicrobial, *Pseudomonas aeruginosa*, Methicillin-resistant *Staphylococcus aureus* (MRSA), Biofilm, Antibiofilm, Extracellular polymeric substances (EPS)

## Abstract

**Background:**

Infected wounds are a major health problem as infection can delay wound healing. Wound dressings play an important part in wound care by maintaining a suitable environment that promotes healing. Silver sulfadiazine dressings have been used to prevent infection in burn wounds. Presently, many commercial silver dressings have obtained FDA clearance.

**Results:**

In this study, we report on a novel silver dressing using microporous aluminosilicate zeolites, termed ABF-XenoMEM. Silver and zinc ions are encapsulated in the zeolite supercages. We show that the silver-zinc zeolite (AM30) alone is effective at inhibiting biofilm formation. The encapsulation protects the silver from rapidly precipitating in biological fluids. We exploit the negatively charged zeolite surface to associate positively charged quaternary ammonium ions (quat) with the zeolite. The combination of the AM30 with the quat enhances the antimicrobial activity. The colloidal nature of the zeolite materials makes it possible to make uniform deposits on a commercial extracellular matrix membrane to develop the final dressing (ABF-XenoMEM). The optimum loading of silver, zinc, and quat on the dressing was found to be 30, 3.7, and 221 µg/cm^2^. Using a colony biofilm model, the activity of ABF-XenoMEM is compared with four well-studied silver-based commercial dressings towards mature biofilms of *Pseudomonas aeruginosa* PAO1 (ATCC 4708) and methicillin-resistant *Staphylococcus aureus* (ATCC 33592). Cytotoxicity of the dressings was examined in HepG2 cells using the MTT assay.

**Conclusion:**

This study shows that the ABF-XenoMEM is competitive with extensively used commercial wound dressings in a colony biofilm model. Nanozeolite-entrapped silver/zinc antimicrobials in association with quat have the potential for application in biofilm-infected wounds and require animal and clinical studies for definitive proof.

**Graphical abstract:**

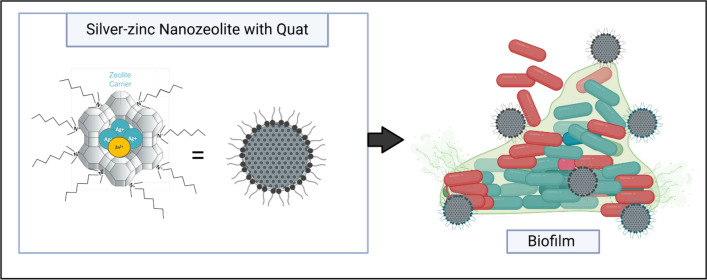

**Supplementary Information:**

The online version contains supplementary material available at 10.1186/s11671-025-04208-8.

## Introduction

Chronic wounds are often associated with vascular, diabetic, and pressure ulcers and time span for chronicity has been defined in the range of 4– 12 weeks [1]. They pose a 1–2% lifetime risk (affect up to 30.9% of the population in developing countries) and lead to reduced quality of life, deaths, and high economic costs [2–6]. In 2014, 15% of Medicare patients in the US had a wound infection, and 4% had surgical site infections [5, 6]. $96.8B is spent on wound care, with about $7.2B for chronic wound care[6].

Biofilm formation is associated with 78% of chronic and 6% of acute wounds [7, 8]. Biofilms are 3D structures enclosed by a matrix of self-secreted extracellular polymeric substances (EPS) [9–11]. Bacteria in biofilms have several defense mechanisms, including the protection offered by the EPS, hypoxia, overexpression of stress genes, and antimicrobial-resistant dormant cells [12, 13]. The bacterial microcolonies within these biofilms have altered phenotypes compared to their planktonic counterparts and display tolerance to host immune defense and administered antimicrobial agents. Systemic antibiotic therapy, the mainstay treatment of bacterial infections, therefore, is of limited efficacy due to the difficulty of the compounds reaching bacteria enmeshed within the biofilm and the greater tolerance of the cells to the antimicrobial compounds [9, 14]. Chronic wound infections are generally polymicrobial in nature [15–17].

Biofilms in chronic and burn wounds can delay the wound-healing process [18]. Thus, approaches to prevent the formation of biofilms, as well as to disrupt biofilms and kill bacteria are necessary. Since biofilms are structurally robust, strategies to remove biofilms involve mechanical and chemical disruption. Mechanical debridement is painful to the patient and can cause damage to healthy tissue and promote the spread of infection [19]. The use of wound dressings to promote wound healing is a common clinical practice.

Silver is effective against both gram-positive and gram-negative bacteria [2]. The silver antimicrobial mechanism of action is mediated through silver ions, which bind to cellular structures and intracellular proteins (N, O, or S functionalities) and bacterial DNA and RNA, interfering with cellular processes. Malfunctions of respiratory chains, cellular toxicity mediated through ROS evolution, as well as structural changes in cell wall can all be mediated by silver ions [20]. Silver has anti-inflammatory effects [15], is anti-angiogenic [21], and influences the immune response [22]. As an effective antimicrobial, silver is helpful for the reduction of infections when used at sub-toxic levels; at higher levels, it can cause discoloration of the skin, known as argyria [23].

The first silver-based material to be used as a dressing to prevent infection in burn wounds was introduced in 1968 in the form of silver sulfadiazine (SSD) [24, 25]. SSD combines silver and the antimicrobial sulfadiazine, applied as an ointment or cream, and been shown to reduce microbial burden [26]. SSD application needs to be changed twice daily, and SSD use is associated with reports of more patient pain [27]. This has led to the introduction of silver dressings with more controlled silver release than SSD dressings and these dressings do not need to be changed as often [28]. Based on the FDA 510 K Premarket Information, there are about 123 different brands of silver wound dressings. Early intervention with silver dressing may decrease biofilm formation, though it is still debated what silver dressing can do if biofilms are already established [29]. Dressings that alleviate infection can minimize the effects of other unfavorable features, such as hypoxia that delay wound healing.

Zeolites are crystalline microporous aluminosilicates with well-defined internal porosities at the sub-nanometer and nanometer scale [30]. These internal cages and channels are accessible to ions and molecules. Zeolites can be synthesized with different morphologies. In this paper, we report on the development of a wound dressing referred to as ABF-XenoMEM with several novel features. To the best of our knowledge, there are no zeolite-based wound dressings. The important aspect of designing new silver dressings is to alter the delivery of silver so that it is not precipitated and can reach deeper into the wounds. Dressings with lower levels of silver to reduce cytotoxicity as well as antimicrobial actives that can penetrate mature biofilms are necessary. In designing this dressing, we have used silver ions trapped in a nanosized microporous aluminosilicate zeolite particle. The hypothesis is that the silver ion trapped in the zeolite is not immediately accessible to constituents of wound fluids, and thus the silver ions are not inactivated by precipitation but can be slowly released over time. Because of the zeolite’s role as a protective encapsulant, lower amounts of silver may still be effective, leading to lower cytotoxicity. In addition, the zeolite makes it possible to colocalize zinc ions alongside the silver ions to improve the antibacterial activity. Further development includes the use of a positively charged quaternary ammonium compound (quat) that can coulombically associate with the negative surface of the zeolite. The purpose of using the quat is to help with the disruption of the biofilm. To make the wound dressing, the zeolite and quat are deposited on a commercial extracellular matrix dressing (XenoMEM™). The antimicrobial properties of the zeolite particles, biofilm inhibition, and examining the number of bacteria in mature biofilms is presented, focusing separately on *Pseudomonas aeruginosa* (PAO1) and methicillin-resistant *Staphylococcus aureus* (MRSA). The study concludes with comparison of the ABF-XenoMEM dressing with four commercial dressings (Procellera™, Acticoat™ 7, Promogran Prisma™ and Aquacel® Ag^+^ Extra™) for killing bacteria in established biofilms. The cytotoxicity of all the dressings was compared using the MTT assay.

## Materials and methods

### Materials

*Zeolite:* Nanozeolite material was prepared and characterized using our published procedures, including structure and porosity [31]. Sodium ion-exchanged nanozeolite served as a control sample (FAU30). Transition metals (Ag^+^ and Zn^2+^) were ion-exchanged into the nanozeolite, resulting in a colloidal suspension, henceforth labeled as AM30. We prepared Ag/Zn zeolites with two compositions. The higher Ag/Zn content (h-AM30) was used for modeling the influence of Zn on the antimicrobial activity (MIC/MBC) and lower Ag/Zn (labeled AM30) was used for all the other experiments, including making the wound dressings. Lower Ag/Zn should have lower cytotoxicity, and thus the motivation for using it. For h-AM30, ion exchange was carried out sequentially with 0.05 M AgNO_3_ and then 0.01 M Zn (NO_3_)_2_.6H_2_O. For preparing AM30, the nanozeolite was ion-exchanged with a solution made up of 0.0125 M AgNO_3_ + 0.0025 M Zn (NO_3_)_2_. 6 H_2_O. To prepare micron-sized powders, AM30 suspension was spray dried using OLT-SP2000 Double Separated Spray dryer from Xiamen Ollital Technology Co. Ltd. using 3.5 wt% of AM30 suspension. Benzalkonium chloride (BZC) was obtained from Sigma Aldrich. Benzalkonium chloride has a formula of C_9_H_13_ClNR, (R = C_8_H_17_ to C_18_H_37_) and is referred here as quat [32]. In the biofilm-suspension experiments (Fig. 5), benzalkonium nitrate was used, made by ion exchange of the BZC with NaNO_3_, as reported in the literature [33]. We refer to the benzalkonium ion as quat throughout this study. XenoMEM™ was obtained from Viscus Biologics Inc. XenoMEM™ is a wound dressing matrix composed of primary type I collagen and additional extracellular matrix (ECM) proteins, and it is derived from the porcine peritoneal membrane. It retains the native extracellular matrix structure and function by exhibiting a connective tissue side and a basal membrane side indicated by the XenoMEM™ embossing.

The materials used for the colony biofilm assay include TSA and LB broths and agar media, PBS, sterile forceps (either autoclaved or flame-sterilized in 70% v/v ethanol), and Cellulose Acetate Membrane Filters (Type 11,106, pore size 0.45 µm; © 2024 Sartorius AG). In addition, 100-mm-diameter sterile Petri dishes, a UV light source (e.g., UVP 8-W multiple-ray laboratory lamp), sterile 15-ml tubes with tightly fitting lids, a vortex mixer, and a VWR ultrasonic water bath were used.

The commercial dressings used in this study are shown in Table 1. These dressings were obtained from the manufacturers of the dressings indicated on the table and were chosen because they have a considerable market share amongst silver-based wound dressings.

**Table 1:** Brief comparisons of the commercial dressings examined in this study

Product	Manufacturer	Silver Content	Description (Ref)
Promogran Prisma™	3 M	Ag: 20 µg/cm^2^	Sterile, freeze-dried composite of 44% oxidized regenerated cellulose (ORC), 55% collagen, and 1% silver-ORC. Silver-ORC contains 25% w/w ionically bound silver [72]
Procellera™	Vomaris	Ag: 900 µg/cm^2^ Zn: 300 µg/cm^2^	Microcurrent-generating antimicrobial wound dressing consisting of a matrix of alternating silver (Ag) and zinc (Zn) dots held in position on a polyester substrate by a biocompatible binder [74]
Aquacel® Ag^+^ Extra™	ConvaTec	Ag: 170 µg/cm^2^	Hydrofiber™ Technology and Ag^+^ Technology – a unique ionic silver-containing, antibiofilm formulation. Two layers of a needle-punched nonwoven fleece of sodium silver CMC fibers enhanced with disodium EDTA and benzethonium chloride, stitched with a high-purity cellulose thread [71, 72]
Acticoat™ 7	Smith & Nephew	Ag: 1700 µg/cm^2^	Two rayon/polyester non-woven inner cores laminated between three layers of nanocrystalline silver (NCS) ‐coated high-density polyethylene mesh, designed to be the barrier against bacterial invasion [70, 71]
ABF-XenoMEM	ZeoVation	Ag: 30 µg/cm^2^ Zn: 3.7 µg/cm^2^ AM30: 660 µg/cm^2^ BZC: 221 µg/cm^2^	Silver zinc nano zeolite and benzalkonium chloride coated on XenoMEM dressing (porcine peritoneal membrane). XenoMEM is a matrix consisting primarily of type I collagen and contains additional ECM proteins such as laminin, elastin, fibronectin, collagen III and IV, and proteoglycans

### Preparation of ABF-XenoMEM

The bare XenoMEM™ sample (5 cm × 5 cm dimension) was weighed first. XenoMEM™ was stretched from four corners with the help of clips for uniform coating (via a syringe) with the basal smooth side (embossed with XenoMEM™) facing down. The first step was to coat XenoMEM™ connective side with AM30 suspension. 3.77% AM30 colloidal suspension was sonicated for 15 min before coating on XenoMEM™. 438 µL of 3.77 wt% AM30 was uniformly spread on XenoMEM™ with the target loading of 660 µg/cm^2^ AM30. The coating was done manually with a syringe in a line-by-line fashion. Eventually, for making commercial samples, an automated method using spray techniques will be required. The coated sample was allowed to dry at room temperature overnight. The second step was to coat AM30-XenoMEM with BZC suspension. 300 µL of 1.85 wt% BZC was uniformly spread on AM30-XenoMEM with the target loading of 221 µg/cm^2^. The dried ABF-XenoMEM is the final sample.

### Characterization

*Dynamic Light Scattering (DLS) and Zeta Potential*: Particle size analysis of FAU30 and AM30 was performed by DLS using Malvern Zetasizer Nano ZS. Nanozeolite and AM30 at 0.1 wt% were prepared by dilution and sonicated for 10 min before measuring DLS. The mixed (zeolite and quat) sample was transferred to a disposable folding capillary cuvette DTS 1070 and zeta potential was measured using the same instrument.

*HRTEM:* The nanozeolite samples for high-resolution transmission electron microscopy (HRTEM) were prepared for drop casting. One drop of 0.01% nanozeolite/water dispersion was loaded on a TEM grid located on a filter paper. After drying, the samples were used for TEM imaging. HRTEM images of nanozeolite were obtained using a FEI Tecnai F20 S/TEM.

*XRD:* Nanozeolite and AM30 suspensions were centrifuged, and the solids were collected and dried in the oven at 100 °C for 12 h. The dried sample was ground into a fine powder. Powder X-ray diffraction (XRD) patterns were obtained using Bruker D8 Advance Diffractometer with Cu Kα radiation with 0.5 divergences, 0.02 step size, and 0.5 s dwell time.

*SEM:* The morphological characterization of XenoMEM™ and ABF-XenoMEM was carried out with Hitachi SU-70 Schottky field emission gun scanning electron microscopy (SEM) with energy dispersive X-ray spectroscopy (EDS) capability for elemental analysis. The wound dressing sample was cut into 1 cm × 1 cm pieces and mounted on a metal stub using sticky carbon tape. The wound sample was coated with conductive material gold to prevent charge build-up on the sample’s surface.

*Elemental analysis of h-AM30*: In a Teflon bottle, a mixture of HF and HNO_3_ (1:1 ratio) was prepared. 10 mL of the acid mixture was mixed with the weighted amount of h-AM30 suspension and allowed to digest zeolite completely. 130 mL of 0.86 M boric acid was used to neutralize HF. DI water was added to make the total volume 160 mL. Silver and zinc loading in h-AM30 were determined using Shimadzu AA-7000 Atomic Absorption Spectrophotometer.

*Elemental analysis of AM30:* Silver and zinc loading on AM30 was determined by Galbraith Laboratory (Knoxville, TN) after acid dissolution using Inductively Coupled Plasma-Optical Emission Spectroscopy (ICP-OES). Before elemental analysis, the entire sample was digested using acid mixture and then analyzed using the ICP-OES method.

*Elemental analysis of SWF:* Silver and zinc loading on AM30 was determined by Galbraith Laboratory (Knoxville, TN) after acid dissolution using Inductively Coupled Plasma-Optical Emission Spectroscopy (ICP-OES). Before elemental analysis, the entire sample was digested using 10% nitric acid, 4% hydrofluoric acid, 4% hydrogen peroxide, and 25% hydrochloric acid and then analyzed using the ICP-OES method.

### Methods

*Silver release into simulated wound fluid (SWF) from ABF-XenoMEM:* Simulated wound fluid was prepared by using 0.2922 g sodium chloride, 0.1680 g of sodium hydrogen carbonate, 0.0149 g of potassium chloride, 0.0139 g of calcium chloride, 1.65 g of bovine albumin, and 50 mL deionized water, as described in the literature [34]. 2 g of SWF was added to the vial, and 2 sets of 2 cm × 2 cm of ABF-XenoMEM (total silver 229.7 µg) were submerged completely in the vial and incubated for 24 h at RT. After 24 h of incubation, the SWF was retrieved, weighed, and stored in a new vial for silver elemental analysis and DLS measurement. The two sets of ABF-XenoMEM were transferred to a new vial containing a fresh batch of 2 g of SWF and the incubation process started. This process was repeated for 7 days, with samples collected daily. SWFs were sent to Galbraith Laboratory for elemental analysis.

*Zeolite release into SWF*: SWF preparation and ABF-XenoMEM incubation method were the same as the silver release experiment. Retrieved SWF (undiluted) were analyzed using the DLS instrument. The zeolite signal (~ 90–100 nm) was normalized to the SWF DLS peak at ~ 10 nm, and the data was compared from Day 1 to Day 7.

*Zeolite and Quat Association*: Various AM30 and quat mixtures were prepared with a fixed AM30 concentration of 1800 ppm and the quat concentration varied from 0, 20, 50, 100, 126, 150, 250, 500, and 1000 ppm. For each of these samples, zeta potential measurements were taken to analyze the zeolite charge variations as a function of the quat concentration.

## Biological methods

### Bacterial strains and media

For the minimum inhibitory concentration (MIC) experiments, *E. coli* BL-21 was cultured in LB (Luria Bertani broth) broth, and MRSA (methicillin-resistant *Staphylococcus aureus*; strain USA300) was cultured in MH2 (Mueller Hinton 2) broth at 37 °C under aerobic conditions for 24 h. The media for growing these bacteria was based on literature [35, 36]. For the remaining biological experiments, *Pseudomonas aeruginosa* (PAO1) (ATCC 4708) and *Staphylococcus aureus* subsp. aureus MRSA (ATCC 33592) were obtained from the American Type Culture Collection (ATCC). PAO1 was grown in LB medium, while MRSA was cultured in tryptic soy broth (TSA), both at 37 °C under aerobic conditions.

### Minimum Inhibitory Concentration (MIC)

MIC procedure described in the literature was followed [37]. Briefly, 50 µl of LB broth was added to each well in a 96-well plate (Agilent Technologies). Nanoparticles (ZnNZ, AgNZ, h-AM30) were sonicated and brought to a concentration of 800 µg/ml in LB broth. 50 µl of nanoparticle (starting concentration 800 ppm) was added to the first well (effectively becoming 400 ppm), mixed 5 times with a pipette, and 50 µl was removed and transferred to the adjacent well. This was repeated for a 10-step dilution array, with 50 µl being discarded from the last wells. The starting inoculum was determined to be 1.27 × 10^8^ cells/ml by OD_600nm_ measurements and was diluted to 5 × 10^5^ cells/ml for the experiments. 50 µl of the cell suspension (5 × 10^5^ cells/ml) was added to each well, halving the concentration of the nanozeolite (except the sterility control). The 96-well round-bottom plate was placed in a 37 °C incubator for overnight incubation. The MIC is being defined as the lowest concentration of the h-AM30 that inhibited the visible growth of the bacteria, estimated visually [37]. MBC (minimal bactericidal concentration) was also determined for the h-AM30 sample [38]. Specifically, 10 µl from each well not showing overnight growth in the presence of the nanoparticle/antibiotic was placed in 100 µl of LB and plated on nutrient agar plates using glass beads to spread on plates. Plates were incubated at 37 °C overnight and assessed for bacterial growth. Colonies, if present, were counted. MH2 media was used with MRSA. The assay was performed with three technical replicates and three biological replicates. MIC was also examined with PAO1 for AM30, and AgFAU30 and ZnFAU30. Figure 3S shows two repeats of the OD600 readings with AM30, ZnFAU30 and AgFAU30. AM30 and AgFAU30 are exhibiting similar MIC (200 ppm), and that ZnFAU30 does not show any inhibitory effect at the concentrations studied.

### Planktonic bacterial killing assay

Cultures of PAO1 were inoculated by scraping a loop of frozen bacterial stock and placing it into LB media. These cultures were grown overnight in a shaking 37 °C incubator. 0.5 µl of overnight culture was added to 4.995 ml fresh LB (5 ml total) to make the logarithmically growing bacterial inoculum, which resulted in 1 × 10^6^ CFU/ml, and allowed to grow for two hours. AM30 particles immediately after being sonicated in the water bath were added to 1-dram vials containing 400 μl of LB media. 100 μl of bacterial inoculum (~ 1 × 10^6^ cells/ml) was then added. Cultures with particles were incubated for 120 min at 37 °C. After the defined period, 50 μl of bacteria with AM30 was placed in 450 μl of broth containing 0.5 wt% thioglycolate and sonicated gently in a water bath for a few minutes. Serial dilutions were made and 100 μl of each was plated on LB agar plates. Plates were incubated overnight at 37 °C and counted the next morning. Killing assay was carried out with three technical replicates, and the experiment was repeated independently three times for biological replicates.

### Biofilm inhibition and crystal violet assay

This assay demonstrates the inhibition of biofilm formation following treatment. 500 µl of water bath-sonicated nanozeolite particles was added to a 1.5 ml microcentrifuge tube containing 400 µl of LB media. Particles were added, and 100 µl of PAO1 bacterial inoculum (~ 1 × 10^6^ cells/ml) was added. Culture was added, and the microcentrifuge tube was vortexed and plated onto a 96-well plate at 200 µl. The final concentration of the nanozeolite FAU30 and AM30 was adjusted to 1.25–100 ppm and the quat at 15. 6 ppm. The starting concentration of the nanozeolite was 1 wt% (estimated gravimetrically by evaporating the water of the nanozeolite suspension). The plate was placed in a 37 °C incubator for 24 h. The bacterial growth was quantified by measuring the optical density at 600 nm (OD_600nm_). The growth media was removed, and the wells were gently washed three times with cold tap water. The biofilms in the wells were then heat-fixed at 70 °C for 1 h [39]. Subsequently, 200 µl of 0.1% crystal violet solution in deionized water was incubated in each well for 15 min. Excess stain was rinsed off with cold water, and the plates were allowed to air dry. To dissolve the crystal violet stain, 200 µl of 33% glacial acetic acid was added to each well, and the biofilm content was indicated by the optical density of 590 nm (OD_590nm_). The Biofilm Inhibition Assay was performed with three technical replicates, and the experiment was repeated independently three times for biological replicates.

### Colony biofilm assay

To conduct the colony biofilm assay, the bacterium of interest was cultured in 5 ml volumes and left to grow overnight until it reached the stationary phase. [40]. Cellulose acetate membranes were then sterilized using UV light for 10 min on each side, positioned approximately 30 cm from the light source. The stationary-phase cultures were diluted in the appropriate medium to about 1 × 10^7^ CFU/ml. A shiny side-up membrane was placed on an agar medium plate without antibiotics, inoculated with 5 μl of the diluted culture, and allowed to dry before incubating the plates upright at 37 °C for 24 h. The membranes were then transferred to fresh agar plates to ensure a continuous nutrient supply for another 24 h. The LB or TSA agar plates contained appropriate nutrients to support bacterial growth. Incubation was carried out for a total of 48 h. After incubation, 100 μl of simulated wound fluid (SWF) was added on top of the biofilm. Commercial wound dressings were then placed on top of the biofilm. Each wound dressing was pre-wetted with 500 μl of sterile water before use, and a sterile glass 60 mm Petri dish was placed on top to provide pressure. Biofilms were treated for 24 h. Following treatment, the membranes and dressings were transferred aseptically to 15-ml tubes containing 10 ml of sterile media neutralizer with 0.5 wt% thioglycolate solution. Samples were vortexed for two pulses of 60 s each and then sonicated for 15 min to ensure the release of all attached bacteria into the wash fluid. The vortexed and sonicated samples were diluted and plated on LB or TSA agar plates, which were then incubated overnight at 37 °C. The average number of colony-forming units (CFU) present in the extracted fluid was counted (reported as CFU/ml considering the entire 10 ml of media used for bacterial extraction) for the dressing and membrane separately (except for ABF-XenoMEM and Promogran Prisma™). The Colony Biofilm Assay was performed with three colonies for each condition, and the experiment was independently repeated three times to ensure biological replicates.

### Cytotoxicity assay

Cytotoxicity assays of the wound dressings were conducted using the MTT assay. The MTT Assay was conducted with three technical replicates per condition, and the experiment was independently repeated three times for biological replicates. Human HepG2 liver epithelial (HB-8065) cells were obtained from the American Type Culture Collection (ATCC, Manassas, VA, USA) and cultured in DMEM (Dulbecco’s Modified Eagle’s Medium, ATCC 30–2002) supplemented with 10% (v/v) fetal bovine serum (FBS) as per the manufacturer’s instructions. We chose HepG2 cells for cytotoxicity evaluation because they are a well-established model for assessing general cytotoxicity and metabolic activity. HepG2 cells, being human liver carcinoma cells, are widely used for cytotoxicity studies due to their ability to metabolize xenobiotics, which provides a comprehensive understanding of the potential toxicity of compounds. Given the role of the liver in detoxification, HepG2 cells offer insights into systemic effects that skin cells may not fully capture. While human skin cell lines are suitable for evaluating direct dermal exposure, our study aimed to evaluate the broader cytotoxic potential of the compounds, making HepG2 a more appropriate choice.

The cells were seeded in triplicate in 96-well plates and incubated at 37 °C in a 5% CO2 atmosphere. The Invitrogen CyQUANT MTT Cell Proliferation Assay Kit was used for the MTT assay. Cells were seeded at a density of 10,000 cells per well in a 96-well plate and incubated for 24 h to allow adherence [39, 41]. A 12-mM MTT stock solution was prepared by adding 1 mL of sterile PBS to a 5-mg vial of MTT and dissolving it by vortexing or sonication. DMEM culture medium on the confluent cells was replaced with 100 µL of Dulbecco’s modified Eagle’s medium (DMEM) colorless with no phenol red, and 10 µL of the 12-mM MTT stock solution was added to each well, including a negative control where 10 µL of the MTT stock solution was added to 100 µL of the medium. Pieces of wound dressings measuring 0.5 cm × 0.5 cm were fully immersed in the medium. The small size of the dressing ensured that it was adequately covered by the culture medium within the 7 mm well diameter. We took care to ensure that all dressings were completely submerged to facilitate uniform exposure of the cells to the dressing materials during the assay. To prevent mechanical detachment of the cells from the bottom of the wells, we optimized the agitation process. The plate was placed on a Corning LSE low-speed orbital shaker and agitated at 10 rpm for 5 min every 30 min (for a total of 4 cycles) before being incubated at 37 °C for 24 h. This gentle agitation ensured that the cells remained adherent and were not disrupted during the process. After treatment, the wound dressing was removed. All but 25 µL of the medium was removed from the wells, and 50 µL of DMSO was added to each well. The mixture was pipetted up and down thoroughly to mix, incubated at 37 °C for 10 min in the dark, mixed again, and the absorbance was read at 540 nm.

### Statistical analysis

To assess the statistical significance of the effect of treatment on bacteria, a Student’s t-test was performed for assays comparing treated bacteria to untreated bacteria. For the biofilm experiments, we compared the ABF-XenoMEM dressing with the commercial dressings. The t-test was conducted using GraphPad Prism version 10.2.3 for Windows (GraphPad Software, Boston, Massachusetts, USA, www.graphpad.com). The following p-value thresholds and corresponding symbols were used to denote significance: ns: p > 0.05 (not significant), *: p ≤ 0.05, **: p ≤ 0.01, ***: p ≤ 0.001, and ****: p ≤ 0.0001. On the figures, p values greater than 0.05 are indicated as ‘ns’, p values less than 0.05 are indicated with one asterisk (*), p values less than 0.01 with two asterisks (**), p values less than 0.001 are indicated with three asterisks (***), and p values less than 0.0001 are indicated with four asterisks (****). Statistical rigor ensures confidence in the reproducibility and reliability of the findings. To account for multiple comparisons, Bonferroni corrections were applied. The corrected significance level was calculated by dividing the original significance level (0.05) by the number of comparisons (m). The BioRender Program was used for generating the TOC graphic.

## Results

### Nanozeolite synthesis, characterization, and silver/zinc ion-exchange

**Fig. 1:**
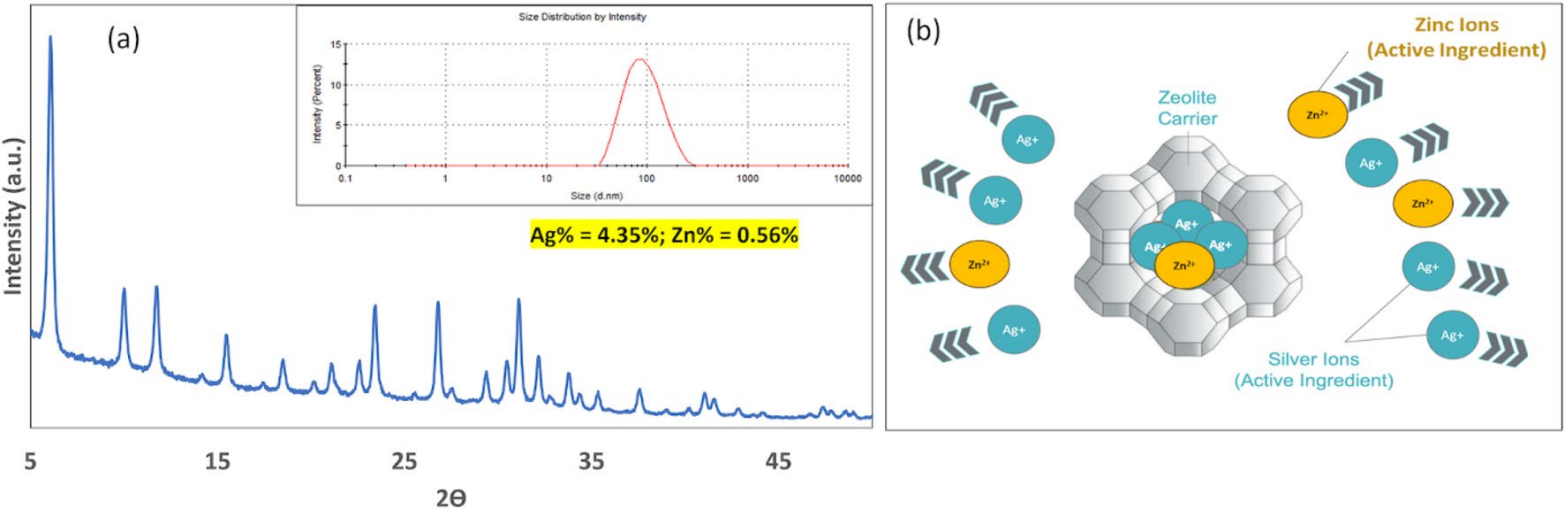
**a** Powder X-ray diffraction of AM30 (inset- dynamic light scattering of AM30 dispersion, (Ag 4.35 wt% % and Zn-0.56 wt%). **b** Schematic of entrapment and release of silver and zinc ions from a zeolite supercage

**Fig. 2:**
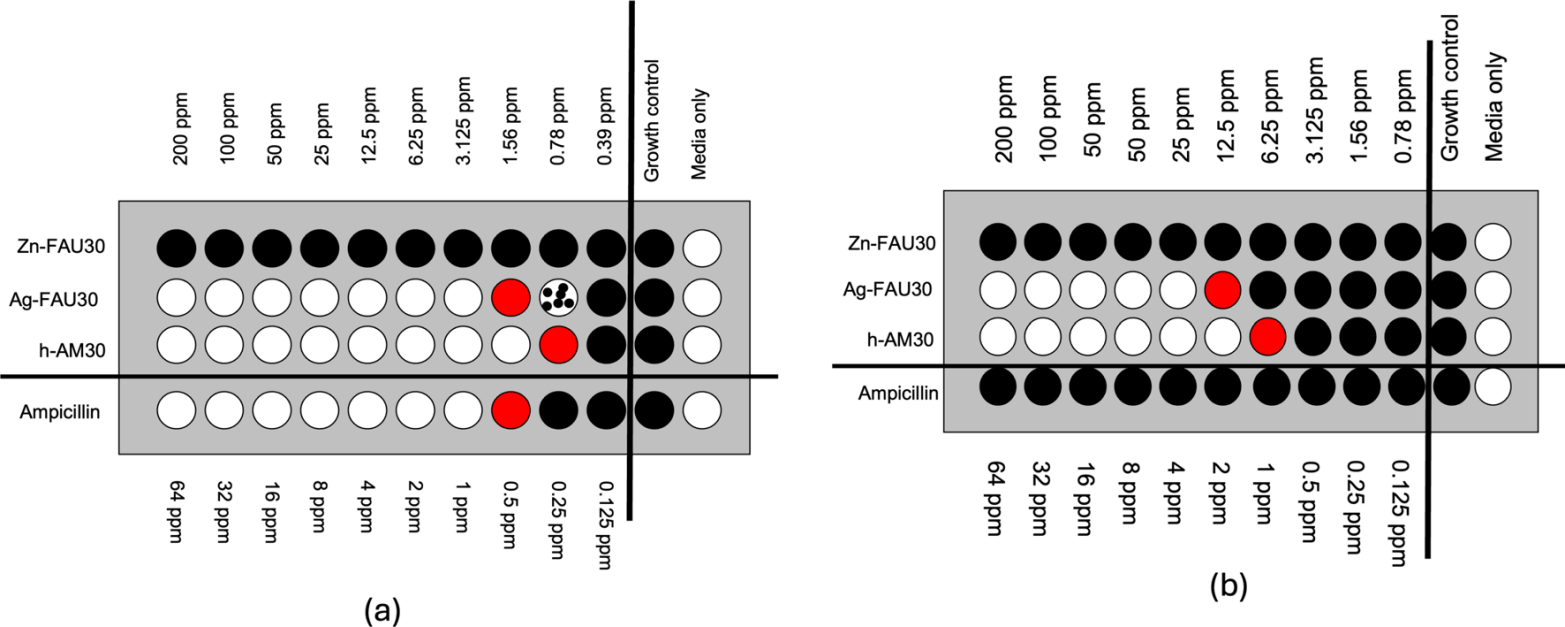
Minimum inhibitory concentration (MIC) comparison of Zn-FAU30, Ag-FAU30, and h-AM30. Black circles indicate complete bacterial growth, partial growth represented by black dots in white, no growth are white circles, and red circles indicate the MIC values (**a**) *Escherichia coli* AM30-MIC 0.78 ppm, AgFAU30-MIC 1.56 ppm, Zn-FAU30 > MIC 200 ppm, ampicillin-MIC 0.5 ppm and (**b**) methicillin-resistant *Staphylococcus aureus* (MRSA), AM30-MIC 6.25 ppm, AgFAU30-MIC 12.5 ppm, Zn-FAU30 > MIC 200 ppm, ampicillin-MIC > 64 ppm

For nanozeolite synthesis, we followed our previously published procedure [31]. Figure 1S (Supplementary Section figures are noted with an S) shows the powder X-ray diffraction and the HRTEM of the sodium ion-exchanged nanozeolite (referred to henceforth as FAU30, which is the control) along with the dynamic light scattering of a diluted suspension. The X-ray diffraction is indicative of the faujasite framework (Fig. 1S top left, compared with the calculated XRD profile for FAU), and the high-resolution transmission electron microscopy (Fig. 1S top right) indicates reasonably uniform particles of average size of 30 nm. The dynamic light scattering indicates an average particle size of ~ 90 nm. It is typical for light scattering to provide larger size numbers than HRTEM [42]. Two silver-zinc zeolites were prepared, h-AM30 with higher concentrations of Ag (17 wt%) and Zn (6.5 wt%) was used for the MIC/MBC experiments, and AM30 with Ag (4.35 wt% %) and Zn (0.56 wt%) for all other experiments. Figure 1a shows the powder X-ray diffraction of AM30 (h-AM30 had a similar XRD profile), and dynamic light scattering of a diluted solution of AM30 (0.1 wt% zeolite). The ion exchange conditions have no effect on the basic faujasite structure and the particle size. The AM30 suspensions are indefinitely stable, and Fig. 2S shows the picture of a 1000 ppm AM30 aqueous suspension after storage for two years. No particles settle out over this period, indicating that the suspension is a stable colloidal solution, which will be relevant in the manufacture of the dressing, as explained below.

### Justification for incorporation of zinc into nanozeolite

**Fig. 3:**
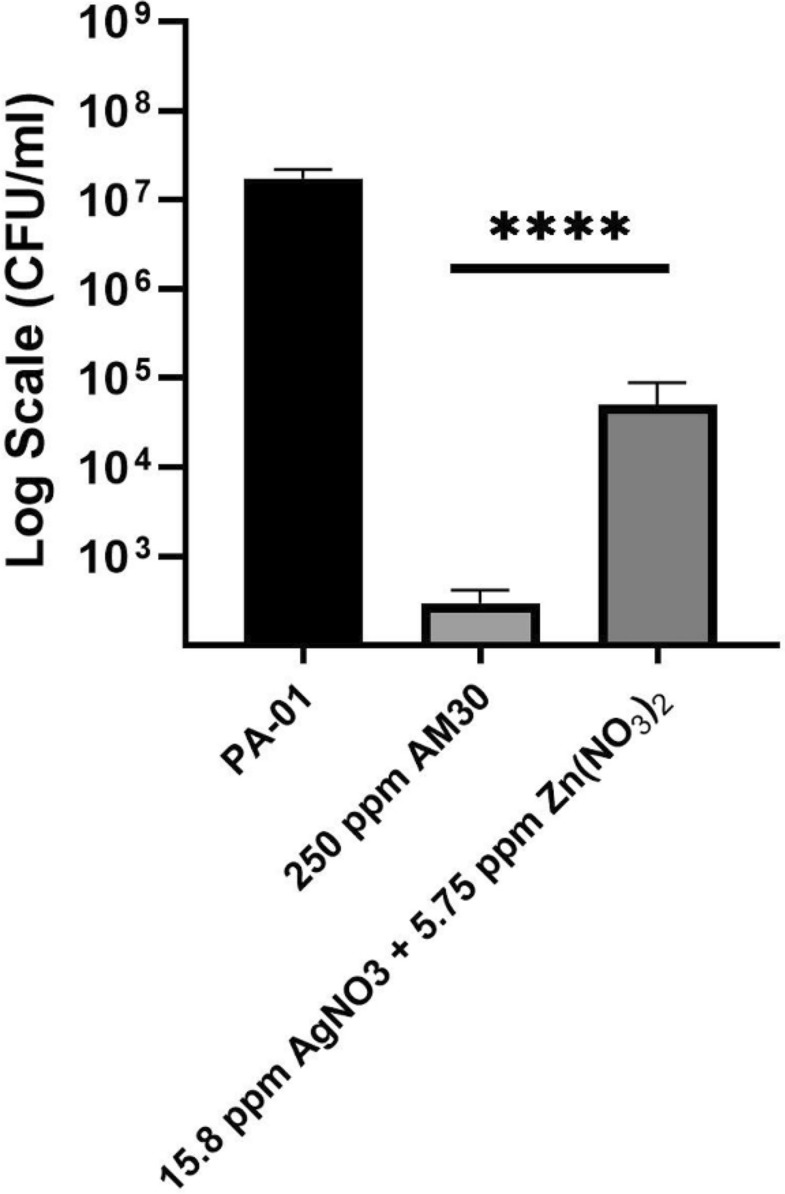
Comparison of the antimicrobial ability of silver ion + zinc ion towards PAO1, when encapsulated in nanozeolite (AM30) and at the same concentration of ions, all samples in LB broth. Exposure time was 2 h at 37 °C. Statistical analysis shows that there is a significant difference between the zeolite-encapsulated and free ions. Student’s t-test, ****p < 0.0001

Silver ion acts as an antimicrobial, and most silver-based wound dressings contain silver ions in the form of salts or metallic silver, often as AgNP [43]. There are reports in the literature that inclusion of zinc ions can increase the antimicrobial activity of the composite, though the exact mechanism is unclear [44]. Thus, the use of zinc will require lower amounts of silver for the same activity. Several bacteria were examined in previous studies with silver-zinc micron-sized zeolites, including *E.coli*, *S. aureus, P. aeruginosa,* and *S*. *cerevisiae* [45]. The zeolite is a unique support [46], as it can incorporate multiple ions within its porous framework [30], thus spatially colocalizing mixtures of ions, we intended to verify that silver-zinc nanozeolite will exhibit similar activity. We examined the MIC for *E.coli* and MRSA, gram-negative and gram-positive bacteria. For the MIC/MBC experiments, h-AM30 was used, the rest of the experiments in this study used AM30. Figures 2a and 2b compare the minimum inhibitory concentration (MIC) values of zinc-exchanged FAU30 (8 wt% Zn), silver-exchanged FAU30 (19 wt% Ag), and h-AM30 (Ag −17 wt% and Zn −6,5 wt%) against *E. coli* and MRSA bacteria, respectively [37, 38]. The procedures we used for noting the bacterial growth in the MIC experiment were visual, as described in Reference [37]. Black indicates growth of bacteria (cloudy) and white indicates no growth (clear), with partial growth shown as black dots in white. The concentration at the transition between growth and no growth indicates the MIC values. For Ag-FAU 30 and AM30, MIC for *E. coli* is 1.56 and 0.78 ppm (same value for MBC) and MRSA MIC for Ag-FAU30 and h-AM30 is 12.5 and 6.25 ppm (same value for MBC), respectively. For both bacteria, the MIC of the h-AM30 is lower by a factor of two as compared to Ag-FAU30, indicating that the inclusion of zinc ions improves the antimicrobial property. Note that for Zn-FAU30, no antimicrobial activity was observed up to concentrations of 200 μg/ml. This experiment served as the justification of the use of zinc in the silver-zinc nanozeolite composition. Since the wound dressing using AM30 has focused on PAO1, we have also done a MIC study comparing AM30 with the silver-exchanged version (AgFAU) and zinc-exchanged version (ZnFAU). Data are shown in Fig. 3S. In the case of PAO1, the inhibitory effect upon inclusion of both silver and zinc in the nanozeolite is not pronounced.

### Development of the AM30 silver-zinc composition

It is well known that silver can cause cytotoxicity [47], so we decided to use lower concentration of silver/zinc in the nanozeolite in the development of the wound dressing. We settled on ion-exchange conditions that resulted in a nanozeolite with Ag-4.35 wt% and Zn-0.56 wt% (AM30). This choice is arbitrary but was a good starting point. Other concentrations of silver and zinc in nanozeolites will also exhibit antimicrobial properties. All experiments henceforth are done with AM30.

### Role of the matrix nanozeolite

Our motivation to use the nanozeolite as a host for the antimicrobial metal ions is because of reports in the literature that silver ions can precipitate in the wound medium and thus be deactivated [12, 15, 48]. Enclosing the ions in the zeolite supercages makes them less available to the environment. To prove this hypothesis, we carried out an experiment with PAO1 planktonic cells in LB broth, where they were exposed to a 250-ppm suspension of AM30 (10.9 ppm Ag^+^. 1.4 ppm Zn^2+^), and similar amounts of free zinc and silver ions, all in broth (5.75 ppm Zn (NO_3_)_2_ (Zn- 1.3 ppm) and 15.8 ppm AgNO_3_ (Ag- 10.0 ppm). After a 2 h exposure, the solution was neutralized with thioglycolate, and the bacteria counted. Figure 3 shows the cell counts. As compared to the control, there was a 5-log_10_ decrease in bacteria counts (CFU/ml) for the AM30, whereas with similar concentration of silver and zinc ions, there was a 2-log_10_ decrease in CFU/ml. LB broth contains proteins, which can precipitate the silver ions in solution, whereas with the zeolite, the antimicrobial ions are held within the porous framework and not immediately available to the surrounding matrix. There were no visible changes in the silver-zinc in the broth sample to indicate precipitation, and not surprising since the samples were quite dilute. The use of other physical methods to confirm the precipitation was not carried out.

### Inhibition of biofilm formation

**Fig. 4:**
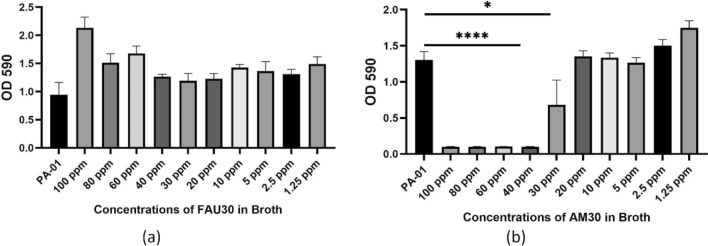
Biofilm inhibition: Comparison of PAO1 inhibition of biofilm formation in LB broth, as measured by the crystal violet assay (a) FAU30 concentrations varying from 1.25–100 ppm, no inhibition of biofilm formation (b) AM30 concentrations varying from 1.25–100 ppm, significant inhibition of biofilm formation at concentrations above 30 ppm. Statistical significance was determined using a t-test. *p < 0.05, **p < 0.01, ****p < 0.0001

The ability of AM30 to inhibit PAO1 biofilm formation was examined using the crystal violet assay [40]. FAU30 was used as the control treatment. Figure 4a shows that with FAU30 treatment, robust biofilms were still formed at all concentrations ranging from 1.25 to 200 ppm. For AM30, the data in Fig. 4b shows that at concentrations greater than 30 ppm, PAO1 biofilm formation was not observed. Thus, we conclude that AM30 can significantly inhibit the formation of PAO1 biofilm. Experiments with PBS were also done and shown in Fig. 4S. The biofilm inhibition takes place at lower concentrations. Concentrations above 5 ppm AM30 are inhibiting growth in PBS, as compared to 30 ppm AM30 for broth.

### Inhibition of biofilm formation of AM30 via association with quat

**Fig. 5:**
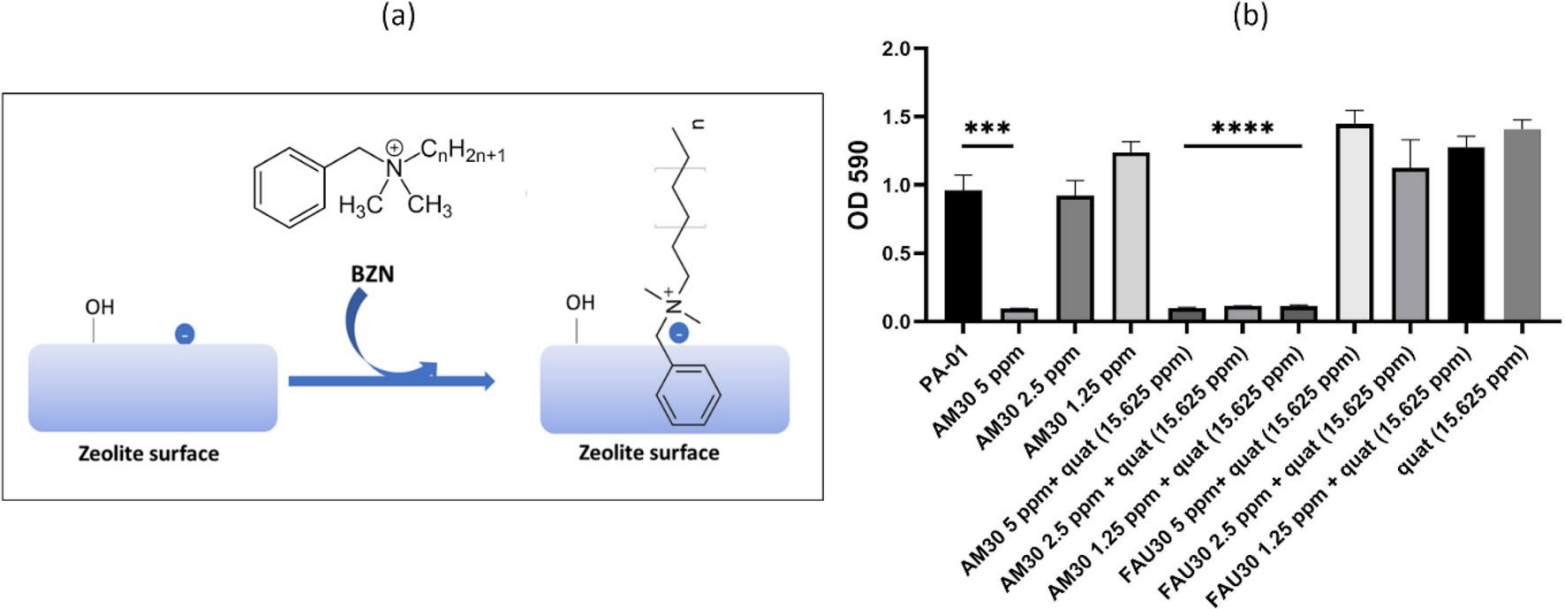
Influence of quat + zeolite on biofilm inhibition. **a** Benzalkonium ion (quat) and its possible association with the negatively charged surface of a zeolite. **b** PAO1 biofilm inhibition by AM30, quat, and AM30 + quat combinations in PBS. AM30 at 2.5 and 1.25 ppm showed no significant inhibition of biofilm formation. FAU30 with quat showed no effect on biofilm formation. Quat at 15.625 ppm showed no inhibition of biofilm formation. Combinations of AM30 at 2.5 and 1.25 ppm with 15.625 ppm quat significantly inhibited biofilm formation. Statistical significance was determined using a t-test. *p < 0.05, **p < 0.01, ***p < 0.001, ****P < 0.0001

Quaternary ammonium compounds, commonly referred to as quats are extensively used as disinfectants [49]. As shown in Fig. 5a, quat (benzalkonium ion) has a positive charge. The surface of the zeolite framework is negatively charged and the association between the zeolite framework surface and cations is well known. The quat is too large to enter the 7.4 Å zeolite supercages and will be coulombically associated with the surface of the zeolite, as schematically represented in Fig. 5a. In order to prove this association, zeta potential measurements were made as a function of the zeolite: quat ratio. AM30 was maintained at a fixed concentration of 1800 µg/ml, and quat concentration varied from 0, 20, 50, 100, 126, 150, 250, 500, and 1000 µg / ml; the zeta potentials were −33.6, −27. −23, −10.4, −4.49, + 0.8, + 10.5, + 20.9, + 28.9 mV, respectively. As expected, the zeolite alone had a negative potential (−33.6 mV) since the surface is negatively charged, and as the quat is titrated in, the potentials become more positive, reflecting the positive charge on the quat, and its increasing association with the surface of the zeolite. Broth was not used in these experiments since zeta potential measurements were not possible in the broth, due to interference from broth constituents.

We examined if zeolite-quat associations can enhance the biofilm-inhibiting activity of AM30. The biofilm-inhibiting activity of AM30 (1.25–5 ppm) with 15.6 ppm of quat was examined with PAO1 in PBS (note that in PBS lower concentrations of AM30 are required to have the antimicrobial effect, Fig. 4S). PBS was chosen instead of broth since the zeta potential experiments that indicate an association between the zeolite and quat were done in water. Figure 5b shows that with AM30 concentrations greater than 2.5 ppm, biofilm formation was inhibited. Inhibition of biofilm formation did not occur with 15.6 ppm quat. The MIC of *Pseudomonas aeruginosa* does vary based on strain, but in general, this bacterium tends to show relatively high resistance to quats. Values for MIC are higher than 50 ppm, with *Pseudomonas aeruginosa (ATCC® 27,853™,* MIC is reported to be 80 ppm [50]. Thus, it is not surprising that at concentrations of 15 ppm quat, we do not observe biofilm inhibition. However, upon mixing the 1.25 ppm AM30 and 15.6 ppm quat, there was significant inhibition of biofilm formation.

### Wound dressings with AM30 and quat

**Fig. 6:**
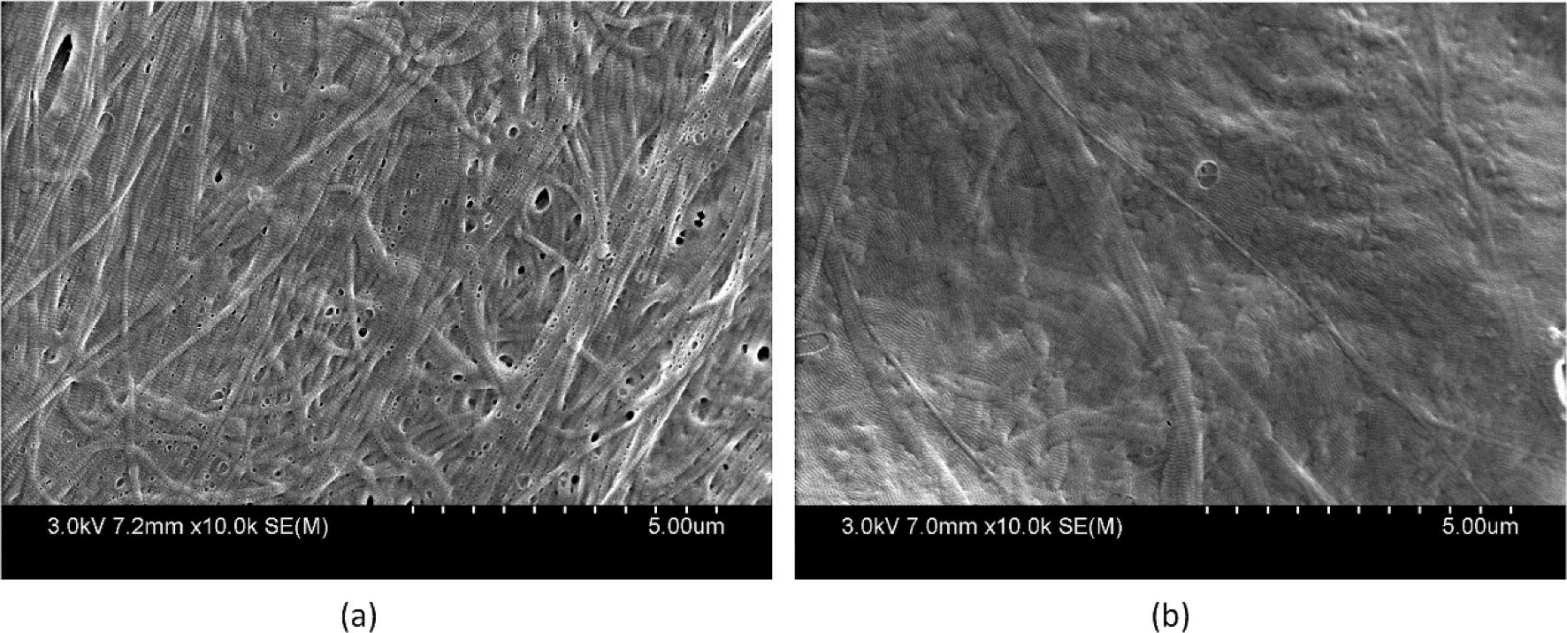
Scanning electron micrograph of the surface of XenoMEM™ (**a**) basal side (**b**) connective side

To design a wound dressing, we needed a suitable matrix on which to deposit the antimicrobials. We chose to use an extracellular membrane matrix, marketed by Viscus Biologics as XenoMEM™. Extracellular matrix (ECM) based wound dressings are used in clinical practice [51, 52]. XenoMEM™ is commercially available through Medline Industries (the product is marketed as Puracol® Ultra ECM). XenoMEM™ is a decellularized, lyophilized, and sterilized porcine peritoneal membrane containing collagen (Types I, III, IV), fibronectin, elastin, laminin, vascular endothelial growth factor, and fibroblast growth factor 2 [53]. Figure 6 shows the SEM of the two sides of the XenoMEM™ dressing, a basal side and a connective side.

**Fig. 7:**
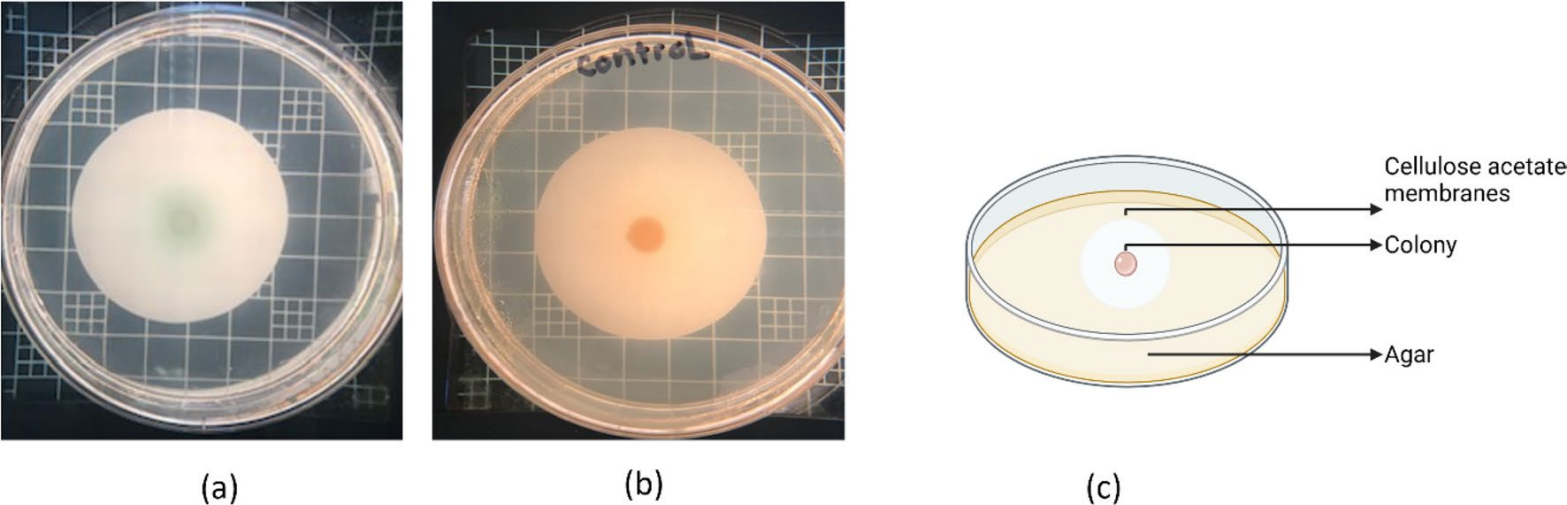
Photograph of the 48 h biofilms grown on the cellulose membrane (**a**) PAO1on LB media (**b**) MRSA on TSB media (**c**) Schematic of the biofilm assembly on membrane and agar (adapted from [40]

**Fig. 8:**
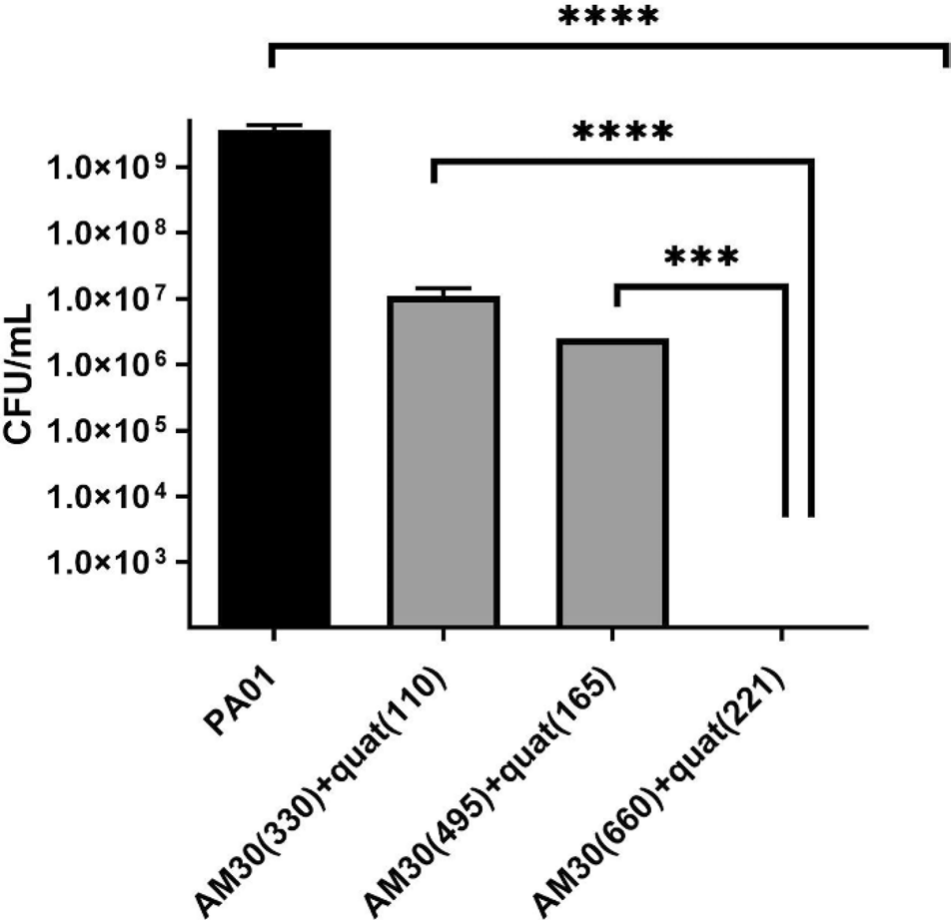
Optimization of concentrations of actives on XenoMEM to create wound dressing. Mature PAO1 biofilms (grown for 48 h) on cellulose membranes were exposed to AM30 + quat at different concentrations on a XenoMEM matrix (concentrations in parentheses in ppm). After 24 h of exposure, the XenoMEM dressing was removed, and bacterial counts on the cellulose membrane (M) were analyzed. The cellulose membrane (M) was treated with media, vortexed, and sonicated, and the bacterial counts in the extract were analyzed (CFU/ml). Statistical significance was determined using a *t*-test. **** represents *p* < 0.0001*** represents *p* < 0.001

To optimize the concentration of the additives, we prepared three samples, XenoMEM™ with AM30 (330 µg/cm^2^) + quat (110 µg/cm^2^), AM30 (495 µg/cm^2^) + quat (165 µg/cm^2^) and AM30 (660 µg/cm^2^) + quat (221 µg/cm^2^). To test the efficacy of these samples, we chose to examine mature PAO1 biofilms grown on a cellulose membrane (48 h growth of biofilm, Fig. 7a). The XenoMEM™ with the additives was placed on top of these biofilms resting on a nutrient agar plate (Fig. 7c), As seen in Fig. 8, the AM30 (660 µg/cm^2^) + quat (221 µg/cm^2^) essentially destroyed the biofilm and with no bacterial counts in the extract, and so we chose the AM30 (660 µg/cm^2^) + quat (221 µg/cm^2^) as our optimized dressing, and henceforth labeled as ABF-XenoMEM. The reason for not increasing the concentration of the antimicrobials any further in the dressing was that both silver and quat are cytotoxic, and cytotoxicity increases with increasing concentrations.

**Fig. 9:**
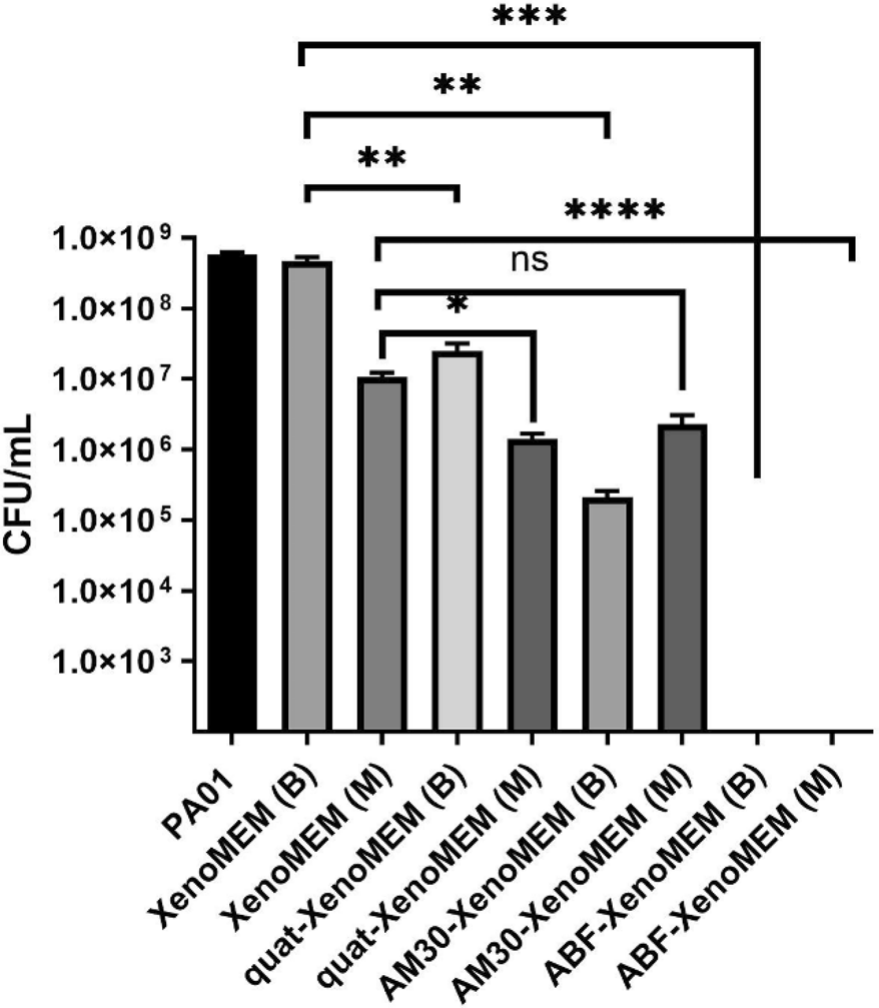
Mature biofilm studies. PAO1 mature biofilms (grown for 48 h) on cellulose (M) were exposed to XenoMEM dressings (B) coated with quat (221 µg/cm^2^), AM30 (660 µg/cm^2^), and AM30 (660 µg/cm^2^) + quat (221 µg/cm^2^) (ABF-XenoMEM) for 24 h. The dressing (B) and the cellulose membrane (M) were treated with media, vortexed, and sonicated, and the bacterial counts in the extract were analyzed (CFU/ml). Statistical significance was determined using a t-test. The p-values are indicated as follows: ****p < 0.0001, ***p < 0.001, **p < 0.01, *p < 0.05, and ns for not significant

The antibiofilm activity of the AM30 + quat as a combination is evident in Fig. 9. Mature biofilms of PAO1 (formed for 48 h) were exposed to XenoMEM™, AM30 (660 µg/cm^2^) on XenoMEM, quat (221 µg/cm^2^) on XenoMEM and ABF-XenoMEM for 24 h, and both the membrane (M) and the dressing (B) were analyzed for resident bacteria. Only in the case of ABF-XenoMEM, the bacteria are absent in the extracts from both the dressing and the membrane. The combination of AM30 + quat is the most effective against mature biofilms.

### Characteristics of ABF-XenoMEM

**Fig. 10:**
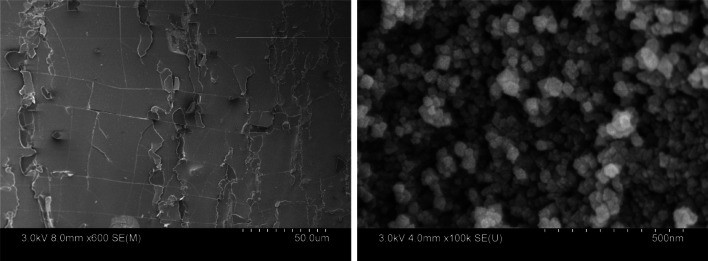
Scanning electron micrograph of ABF-XenoMEM at two magnifications

SEM of ABF-XenoMEM is shown in Fig. 10, and at low resolution appears to be uniformly covering the XenoMEM™. At higher resolution, the zeolite particles can be seen distributed over the XenoMEM™. With the nanoparticle coating, the coating appears to be robust, and particles do not fall off from the dressing upon handling. Figure 5S shows the elemental map (by EDS) for silver and appears to be uniformly distributed.

There are numerous studies on micron-sized silver zeolites, including availability from commercial sources. We want to point out that such zeolites would not function well as additives for wound dressings. Two aspects of the AM30 makes it appealing for this application. First is the indefinite stability of AM30 in aqueous suspensions (Fig. 2S), which makes it easy to generate uniform coatings on surfaces by spray/drop coat methods ( micron-sized particles will settle out). Second, the use of micron-sized zeolites (formed by spray drying of AM30, Fig. 6S) as coatings on the XenoMEM™ are unstable and fall off (Fig. 6S).

For the dressing to be effective, the zeolite particles will need to be released into the wound fluid. To simulate this release, ABF-XenoMEM was placed in SWF, and DLS of the suspension was carried out to get a measure of how much zeolite is being released as a function of time. Figure 7S shows this data, and it appears that most of the release of the particles in the SWF is occurring between days 1–4. To get a quantitative estimate of the release, an elemental analysis of the extracts was carried out by ICP-OES. It was found that silver release was complete within 4 days, with 39.4% at end of day 1 (61 ppm in the volume of SWF analyzed), 30.7% end of day 2 (44 ppm in the volume of SWF analyzed), 19.8% at end of day 3 (29 ppm in the volume of SWF analyzed), and 10.1% at end of day 4 (15 ppm in the volume of SWF analyzed). Amounts of silver in samples recovered on days 5–7 were below the detection limits of the instrument. The exact nature of the bonding forces between the nanozeolite and ECM layer that leads to stable layers has not been explored in this study, but it is sufficient to have an extended release of the nanozeolite over at least a period of four days (Fig. 7S).

The choice of the matrix of XenoMEM™ was made with the rationale that once the biofilm is penetrated by the quat + zeolite and bacteria killed, then the ECM matrix can assist in wound healing. XenoMEM™ has been shown to assist wound healing [53].

### Comparison of ABF-XenoMEM with commercial silver dressings

Four commercial dressings were chosen for comparison with ABF-XenoMEM. In Table 1, we outline the specifics of these dressings.

**Fig. 11:**
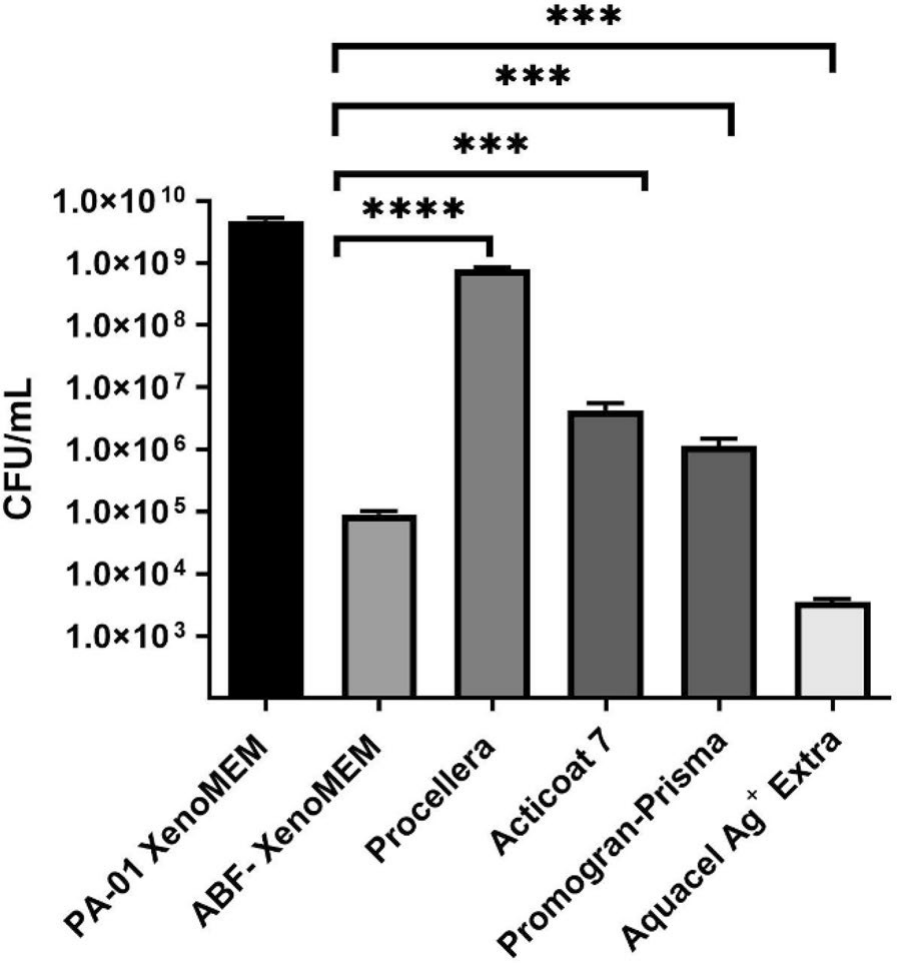
Comparison of commercial wound dressings and ABF-XenoMEM for biofilm studies. PAO1 biofilms were grown on cellulose membrane (M) for 48 h and exposed to ABF-XenoMEM, Procellera, Acticoat 7, Promogran Prisma, and Aquacel Ag + Extra for 24 h (B). The dressing and membrane were separated (except for ABF-XenoMEM and Prisma). The dressing (B) and the cellulose membrane (M) were treated with media, vortexed, and sonicated and the bacterial counts in the extract were analyzed (CFU/ml). The data plots the sum of CFU/ml on B and M. For CFU/ml on B and M separately, see Fig. 8S. Statistical significance was determined using t-tests. The p-values are indicated as follows: ****p < 0.0001, ***p < 0.001, **p < 0.01, *p < 0.05, and ns for not significant

**Fig. 12:**
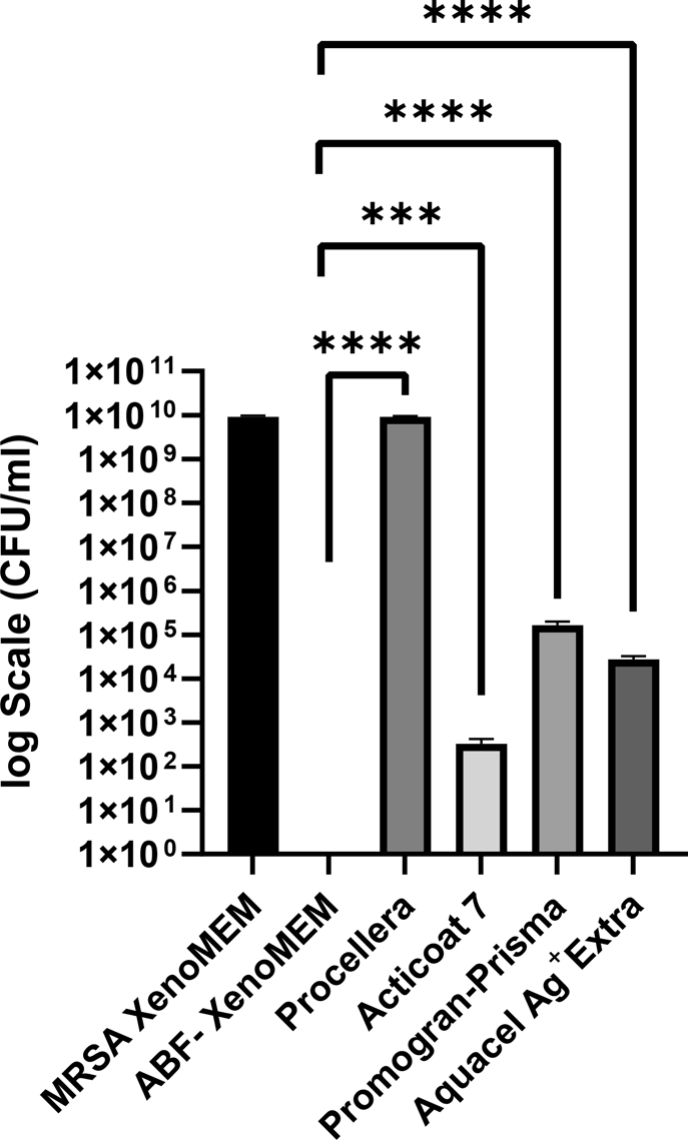
Comparison of commercial wound dressings and ABF-XenoMEM for biofilm studies. MRSA biofilms were grown on cellulose membrane (M) for 48 h and exposed to ABF-XenoMEM, Procellera, Acticoat 7, Promogran Prisma, and Aquacel Ag + Extra for 24 h (B). The dressing and membrane were separated (except for ABF-XenoMEM and Prisma). The dressing (B) and the cellulose membrane (M) were treated with media, vortexed, and sonicated and the bacterial counts in the extract were analyzed (CFU/ml). The data plots the sum of CFU/ml on B and M. For CFU/ml on B and M separately, see Fig. 9S. Statistical significance was determined using a t-test. *p < 0.05, **p < 0.01, ****p < 0.0001

*Biofilm Studies:* To compare the different dressings, we exposed mature biofilms (48 h growth) to the different dressings. Figure 7 shows photographs of the PAO1 and MRSA biofilm grown for 48 h on the cellulose matrix. A change from previous experiments (Figs. 8,9) is that we put a layer of SWF between the dressing and the biofilm to better represent a wound environment, as reported in previous studies [12, 54]. With the ECM matrix dressings, ABF-XenoMEM and Promogran Prisma™, the dressing could not be completely separated from the cellulose layer, and both were analyzed together for resident bacteria (for ABF-XenoMEM this was not the case without SWF (Fig. 9)). Figure 11 shows the results at the conclusion of 24 h exposure to the different wound dressings for PAO1 biofilms (the bacteria on the dressing and membrane are added, the bacteria on the individual dressing and membrane are shown in Fig. 8S). All the dressings have a significant effect on reducing the bacterial load for the gram-negative bacterial biofilm. Aquacel® Ag^+^ Extra™ seems to perform the best in terms of bacterial reduction, then ABF-XenoMEM, which was effective, followed by the Promogran Prisma™, Acticoat™ 7 and Procellera™. Figure 12 shows the results at 24 h exposure of the wound dressings for mature MRSA biofilms (48 h) (membrane and dressing bacteria are added together, Fig. 9S for bacterial counts analysis of the extracts from the dressing and membrane separately). Against MRSA, only the Procellera™ did not exhibit a significant decrease in the bacterial load. The ABF-XenoMEM performed significantly better than the other dressings against the gram-positive MRSA biofilm, followed in order by Acticoat™ 7, Aquacel® Ag^+^ Extra™ and Promogran Prisma™.

#### Cytotoxicity

**Fig. 13:**
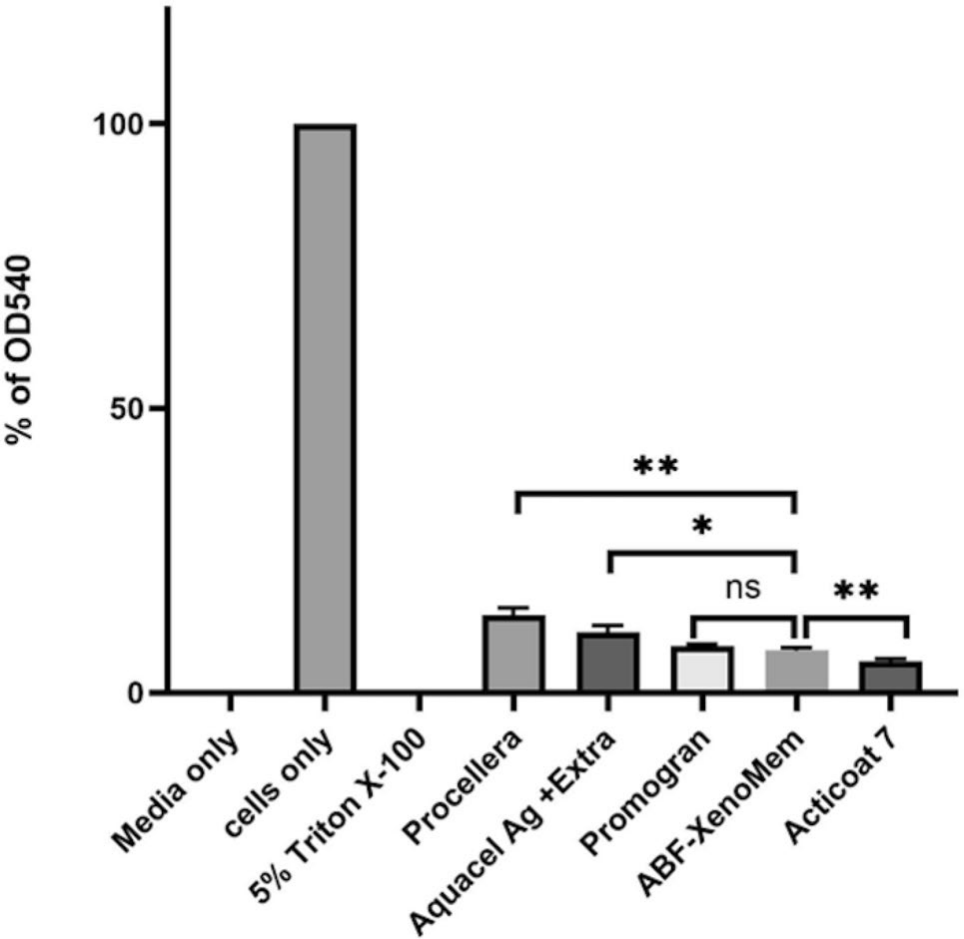
Comparison of cytotoxicity toward confluent HepG2 Cells (MTT Assay). This figure presents the cytotoxic effects of commercial dressings compared with ABF-XenoMEM on HepG2 cells. Statistical significance was determined using a t-test. Symbols indicate levels of significance: ns (not significant), *p < 0.05, **p < 0.01

The dressings were compared for their cytotoxicity towards HepG2 cells using the MTT assay (Fig. 13) [55]. Consistent with the literature on silver dressings, all the dressings were cytotoxic, with ~ 10% or lower cell viability. Within statistical significance, ABF-XenoMEM is less cytotoxic than Acticoat™ 7, but more so than Procellera™ and Aquacel® Ag^+^ Extra™ and no significant difference in cytotoxicity as compared to Promogran Prisma™.

## Discussion

In this section, we focus on the novel structural aspects of the new dressing ABF-XenoMEM, and its comparison with commercial dressings towards mature PAO1 and MRSA biofilms.

### Structural aspects

In complex biological media, the amount of silver necessary to have an antimicrobial effect increases significantly [25, 56, 57]. Proteins and membranes in biological media form complexes with silver ions [58], and silver deposits are found in clinical wound exudates from patients with venous ulcers [59]. Nutrient media decreases the bioavailability of silver by 2 orders of magnitude as compared to water/PBS and the level of silver is fivefold lower in biological fluids as compared to nutrients [12, 56, 60]. The use of zeolite offers protection from precipitation of the encapsulated silver ion versus silver ion in the free form. It has been noted that even with sustained delivery of silver in chronic wounds, residual infections are still present, indicating that silver may not be reaching the interior of the wounds or that biofilms prevent the antimicrobial effect [58]. Though not proven in this study, it is possible for the colloidal nanozeolite to be stabilized due to the formation of a protein corona around it [61].

A second aspect is the Coulombic association of zeolite with a quat. Such systems for solute/drug extraction has been reported [62]. Studies of zeolite quat composites shows similar trends as reported here, with the negative charge on the zeolite being compensated with increasing amounts of quat, and finally reaching positive potentials, indicating charge reversal and multilayer formation as the quat covers the zeolite surface [62, 63]. However, the quat-AM30 being coulombically associated is still a hypothesis in real wound fluids, since we have not studied how the association changes in a complex biological fluid with other charged species present. The data does demonstrate that the combination of the zeolite and the quat is most efficient at killing the biofilm bacteria (Fig. 9). There is a commercial dressing with quat (benzalkonium chloride) dispersed in polyethylene glycol base (1.44 mg quat in 440 mg of PEG, sodium citrate, and citric acid) [64], that can be compared to the quat-XenoMEM (221 ug/cm^2^ quat) in Fig. 9. We observe a reduction of 2-log_10_ CFU/ml in PAO1 (48 h) biofilm in both dressing and membrane after treatment with quat-XenoMEM as compared to XenoMEM. Several modifications of the quat-PEG dressing (all similar levels of quat) were tested [64], two of these performed similarly with the quat-XenoMEM shown in Fig. 9, but with one of the quat-PEG formulations, the formation of PA biofilm (24 h) was completely disrupted, the paper claims that there is some other proprietary material in this dressing [64]. Another study found the quat-PEG commercial dressing exhibited a reduction of 4-log_10_ decrease in CFU, with the control having 8-log_10_ CFU for PAO1 biofilms grown in a porcine explant model [65].

### Comparison with commercial dressings

Bacteria in biofilms exhibit considerable resistance to antimicrobials as compared to these same bacteria in the form of planktonic cells [14, 66]. With *S. epidermidis* biofilms, AFM studies show that 50 ppb of silver can destabilize the biofilm matrix [67]. Silver-containing wound dressing was found to kill all bacteria in *Pseudomonas aeruginosa, Enterobacter cloacae, Staphylococcus aureus* biofilms, as well as in mixed bacterial biofilms within 48 h [68]. Another study shows that the maturity of the biofilms was relevant to the extent of silver action [69]. The susceptibility of *Pseudomonas putida KT2440 biofilms* toward AgNP decreased as the maturity of biofilms increased [69]. Upon removal of the EPS layer, the biofilms became more susceptible to the silver, and it was suggested that AgNP transport through biofilms may be limited for mature biofilms [69]. Considerable effort has gone into designing dressings that can disrupt the biofilm such that the bacteria become accessible, and more planktonic-like so that the antimicrobial silver can destroy the bacteria. We have chosen four such commercial dressings and discuss them in detail below.

*Structural Aspects of Commercial Dressings:* Table 1 is a brief comparison of the dressings in this study. Acticoat™ 7 has silver in the form of Ag nanoparticle (instead of silver ions) deposited on a high-density polyethylene mesh. It has the highest amount of silver (1700 µg/cm^2^) amongst the dressings examined. A rayon/polyester non-woven inner core is laminated between the three layers of the Ag NP-polyethylene mesh [70, 71]. AgNP were reported to be better prophylaxis of infection as compared to silver ion dressings [15], the nanoparticles have the potential to reach deeper into biofilms [9]. Aquacel® Ag^+^ Extra™ contains ethylene diamine tetra acetic acid (EDTA), benzethonium (BT) chloride, and silver ion (170 µg/cm^2^) in a hydrofiber matrix [71, 72]. The EDTA is added to sequester ions that help in the structural integrity of the biofilm and the surfactant BT can impact the biofilm architecture [18, 73]. Procellera™ was chosen because it contained both silver (900 µg/cm^2^) and zinc (300 µg/cm^2^) [74]. Procellera™ is made up of alternating metallic silver and zinc dots held by a biocompatible binder on a polyester substrate. In the presence of a conducting medium, there is a voltage generated between the two metals of 0.5–0.9 V, with silver acting as the positive terminal. The proposed mechanism of antimicrobial action is that the negatively charged planktonic bacteria are attracted to the positive charge on the silver electrode, and the ensuing contact promotes the killing of the bacteria. Promogran Prisma™ was chosen since it uses an ECM matrix (as does ABF-XenoMEM), and has the lowest amount of silver ion (20 µg/cm^2^) amongst the dressings examined [72]. ABF-XenoMEM is made by depositing nanoparticles containing silver ion (30 µg/cm^2^), zinc ion (3.7 µg/cm^2^), and quat (221 ug/cm^2^) onto an ECM matrix. ABF-XenoMEM has silver at a slightly higher concentration of silver than Promogran Prisma™ (30 µg/cm^2^ versus 20 µg/cm^2^).

As noted in the SEM micrographs of Fig. 10 and the elemental maps of Fig. 5S, the silver is distributed uniformly on the ECM matrix in ABF-XenoMEM. This contrasts with the silver distribution of Aquacel® Ag^+^ Extra™ and Acticoat™ 7. With Aquacel® Ag^+^ Extra™, SEM shows that the silver ions reside in the background behind the raised stitching, and a lack of antimicrobial effect was noted in specific areas of the dressing due to the dead space [22, 48]. In Acticoat™ 7, isolated silver nanoparticles are observed in the SEM, distributed along the polyethylene net [48].

*Mature biofilm studies:* We compared ABF-XenoMEM with four leading commercial silver-based wound dressings for their ability to reduce the bacteria in mature biofilms of PAO1 and MRSA (Figs. 11 and 12, Figs. 8S and 9S). Our procedure was consistent across all five dressings. Biofilms were grown on cellulose membranes for 48 h, covered with SWF (to mimic the presence of wound fluids), and then exposed to the dressings for 24 h. The arrangement was such that biofilm bacteria could interact with the dressing directly as well as with antimicrobials released into the SWF [12, 56, 60]. The dressings and the membrane were analyzed for resident bacteria (except for Promogran Prisma™ and ABF-XenoMEM, where the membrane and dressing were stuck in places and could not be cleanly separated). There have been many in vitro biofilm studies using similar methodology on the commercial membranes reported in the literature and they provide a benchmark against the data that we observe and form the basis of this discussion. This type of colony biofilm model is very typical as the first experiment with new dressings. It gives an idea of the comparative performance, but conclusions drawn from such experiments as to the performance of the dressings in dealing with real infected wounds cannot be drawn.

ABF-XenoMEM: Twenty-four-hour exposure of a 48 h PAO1 biofilm on a cellulose membrane to ABF-XenoMEM resulted in a reduction of 4.6-log_10_ CFU/ml (compared to the control sample at 9.6-log_10_ CFU/ml) in the extracts from the membrane and the dressing (note that the result is different from Fig. 9 and is possibly due to the inclusion of the SWF in these experiments). With MRSA, no detectable bacteria were remaining on the membrane plus the dressing, thus a reduction of 9.8-log_10_ CFU/ml relative to control. With both these experiments, the dressing could not be completely separated from the membrane, and both were analyzed together.

Acticoat™ 7: In the present study, this dressing performed better with MRSA biofilms, there was a reduction of 7.3-log_10_ CFU/ml (bacterial counts in the extract) relative to control of 9.8-log_10_ CFU/ml, whereas, with PAO1, there was a reduction of 3-log_10_ in CFU/ml (in the extract) relative to control of 9.6-log_10_ CFU/ml (bacterial counts in CFU/ml in the extracts from the membrane and dressing are added). Figures 8S and 9S show the bacterial counts in CFU/ml in the two extracts for the cellulose membrane and dressing, respectively. Literature studies with Acticoat™ 7 provide a comparison with data shown in Figs. 11 and 12. In a mature mixed-species PA + MSSA biofilm established in a Drip Flow Biofilm Reactor for 3 days on hydroxyapatite-coated slides, reductions of 3.42-log_10_ CFU/cm^2^ (statistically significant) for MSSA and 1.3- log_10_ CFU/cm^2^ for PA were noted for Acticoat™ 7 (controls were between 9–10 log_10_ CFU/cm^2^) for a 24 h treatment [75]. In another study [12], for PA biofilm there was a 4.2-log_10_ reduction in CFU/peg (control 7.6-log_10_ CFU/peg), and with MRSA biofilm there was a 4.6-log_10_ reduction in CFU/peg (control 6.2-log_10_ CFU/peg) at 24-h mark with Acticoat™ 7. With PA, the viable cells on dressing were 1.2-log_10_ CFU/cm^2^, and with MRSA cells 1.8-log_10_ CFU/cm^2^ at 24-h mark [12]. The higher activity against *S. aureus* in both studies is consistent with our observation. With biofilms grown on gauze, Acticoat™ 7 was found to be ineffective for both MRSA and PA biofilms (grown for 48-h), and not consistent with our observation [73]^.^

Aquacel® Ag^+^ Extra™: In the present study, this dressing performed better against PAO1 biofilms, showing a reduction of 6.4-log_10_ CFU/ml decrease relative to control of 9.6-log_10_ CFU/ml and with MRSA, a reduction of 5.3-log_10_ CFU/ml relative to control of 9.8-log_10_ CFU/ml (bacterial counts in CFU/ml in the extracts from the membrane and dressing are added). Literature reports with Aquacel® Ag^+^ Extra™ against MRSA biofilm (48 h) found a reduction of 2-log_10_ CFU (control 10 log_10_ CFU) after 24 h exposure, and for PA biofilms (48 h) a reduction of 6-log_10_ CFU (control 10 log_10_ CFU) was observed after 24 h [73]. Results showing significantly better activity towards PA biofilms are consistent with the present study. In another study using a colony drip-flow reactor with younger PA biofilms (24 h), 24 h exposure to Aquacel® Ag^+^ Extra™ exhibited a reduction of 2.6-log_10_ CFU/ml relative to control gauze (9.24-log_10_ CFU/ml); however, for PA biofilms that were matured for 72 h, the dressing brought about a decrease of only 0.4-log_10_ CFU/ml (control gauze 9.2-log_10_ CFU/ml) [72]. In another CDC reactor biofilm model study, 72 h *Staphylococcus aureus* (SA) and PA biofilms were exposed to Aquacel® Ag^+^ Extra™ for 24 h, no bacteria were recovered for both biofilms (a reduction of 4.3-log_10_ CFU/ml compared to control for SA and a reduction of 6.4-log_10_ CFU/ml for PA) [76]. In a different study, SA and PA biofilms were grown on the Whatman cyclopore circular membrane on nutrient agar plates for 24 h to form colony biofilms. Treatment with Aquacel® Ag^+^ Extra™ led to a reduction of 1.2-log_10_ CFU for SA (control 9.2-log_10_ CFU) in 24-h, whereas for PA, no change was observed at 24 h (the study was continued for 5 days, and activity towards SA biofilms were superior that PA biofilms), inconsistent with our study [22].

Procellera™: In the present study, this dressing did not perform well relative to the others, with a reduction of 1-log_10_ CFU/ml following treatment of PAO1 biofilms relative to the control of 9.6-log_10_ CFU/ml and no change relative to the control of 9.8-log_10_ CFU/ml for MRSA (bacterial counts in CFU/ml in the extracts from the membrane and dressing are added). In the literature, the dressing was found to be effective over a 24-h period against killing many planktonic bacteria isolated from clinical wounds [77]. Using a colony biofilm reactor, clinical wound isolates were introduced onto cellulose filter membranes, bacteria were circulated for 72 h and the resident bacteria on the membrane and dressing were analyzed. A reduction of 2-log_10_ CFU/ml was noted for PA (with blank polyester control of 8-log_10_ CFU/ml) and a reduction of < 1-log_10_ CFU/ml decrease for SA with 6-log_10_ CFU/ml for control gauze. This procedure is slightly different from what is being practiced in this paper; however, the extent of bacterial decrease for PA and MRSA biofilms following treatment (1–2 log_10_ CFU/ml) in this study were of similar levels as the biofilm reactor study [78].

Promogran Prisma™: In the present study, a reduction of 3.6-log_10_ CFU/ml relative to a control of 9.6-log_10_ CFU/ml was observed for treated PAO1 biofilms, and a reduction of 4.8-log_10_ CFU/ml for MRSA biofilm relative to the control of 9.8-log_10_ CFU/ml (extracts were prepared from the dressing and membrane combined). From a study using a colony drip-flow reactor, PA biofilms were grown for 24 h, and upon 24 h exposure to dressing, a reduction of 2.77-log_10_ in CFU/ml relative to control gauze with 9.24-log_10_ CFU/ml was observed. These results for PA are consistent with our observations [72].

### Cytotoxicity

Silver toxicity towards prokaryotes and eukaryotes is well studied [43, 79]. Cytotoxicity towards HepG2 cells as evaluated by the MTT assay was quite pronounced for all the dressings (~ 5–10% cell viability). Within statistical significance, Procellera™ and Aquacel® Ag^+^ Extra™ were less cytotoxic than ABF-XenoMEM. (Fig. 13). Acticoat™ 7 with the highest silver content shows the most pronounced cytotoxicity and was significantly more toxic than ABF-XenoMEM. A previous study reported that extracts from Acticoat™ 7 and Aquacel® Ag^+^ Extra™ exhibited comparable toxicity towards keratinocytes by MTT assay [48]. In designing ABF-XenoMEM, we attempted to keep the silver concentration low so as not to disrupt the healing process. However, the inclusion of quat increases the cytotoxicity as noted with quat-PEG dressings (63).

## Conclusion

The new wound dressing ABF-XenoMEM proposed in this paper has the following characteristics: storage of the silver in nanozeolite so that it does not precipitate in the wound fluid, exploiting the microporous nature of the zeolite to spatially co-locate zinc ion with the silver ion to enhance the antimicrobial activity of silver, the inclusion of positively charged quats with the negatively charged zeolite to promote the anti-biofilm activity of the dressing, and extended release of the antimicrobial silver over 4 days. Overall, we can conclude that the ABF-XenoMEM is competitive with the commercial dressings in decreasing PAO1 and MRSA bacteria encapsulated in biofilms in an in vitro colony biofilm model with each bacteria separately and comparable in cytotoxicity with commercial wound dressings. In vitro results are a promising start for this new type of wound dressing and are a common practice with new wound dressings. Future studies will need to focus on polymicrobial biofilms, ex-vivo assays, animal models, and clinical studies to demonstrate the true potential of this nanozeolite dressing for wound healing of biofilm-colonized wounds.

## Supplementary Information

Additional file1

## Data Availability

The data that support the findings of this study are available from the corresponding authors, Prof. P. K. Dutta (dutta.1@osu.edu) and Prof. M. L. van Hoek (mvanhoek@gmu.edu), upon reasonable request. Any additional information, including protocols and raw datasets, can be accessed by contacting the corresponding authors.

## Abbreviations

MRSA: Methicillin-resistant *Staphylococcus aureus*
PAO1: *Pseudomonas aeruginosa* O1
SA: *Staphylococcus aureus*
FAU30: Nanozeolite
AM30: Silver ion + zinc ion nanozeolite
quat: Quaternary ammonium ion
ABF-XenoMEM: Silver-zinc nanozeolite + quat on XenoMEM™ dressing
SWF: Simulated wound fluid
B: Wound dressing
M: Cellulose membrane
