# Supplementary material for: Activity of silver-zinc nanozeolite-based antibiofilm wound dressings in an in vitro biofilm model and comparison with commercial dressings
Source: Discov Nano. 2025 Feb 11;20(1):26. doi: 10.1186/s11671-025-04208-8 (PMC11813856; doi:10.1186/s11671-025-04208-8)
Supplement: Supplementary file 1 — Additional file1 [file 11671_2025_4208_MOESM1_ESM.docx]

**Supplementary Section**

**Activity of silver-zinc nanozeolite-based antibiofilm wound dressings in an *in vitro* biofilm model**

Sarah Abdulaziz Alobaid^1^, Sweta Shrestha^2^, Morgan Tasseff^1^, Bo Wang^2^, Monique Van Hoek^1,*^, Prabir K. Dutta ^2,3,*^

^1^School of Systems Biology, George Mason University, Manassas, VA, USA

^2^Zeovation Inc., Columbus, OH, US.

^3^Department of Chemistry, The Ohio State University, Columbus, OH, US.

Supplementary Figures


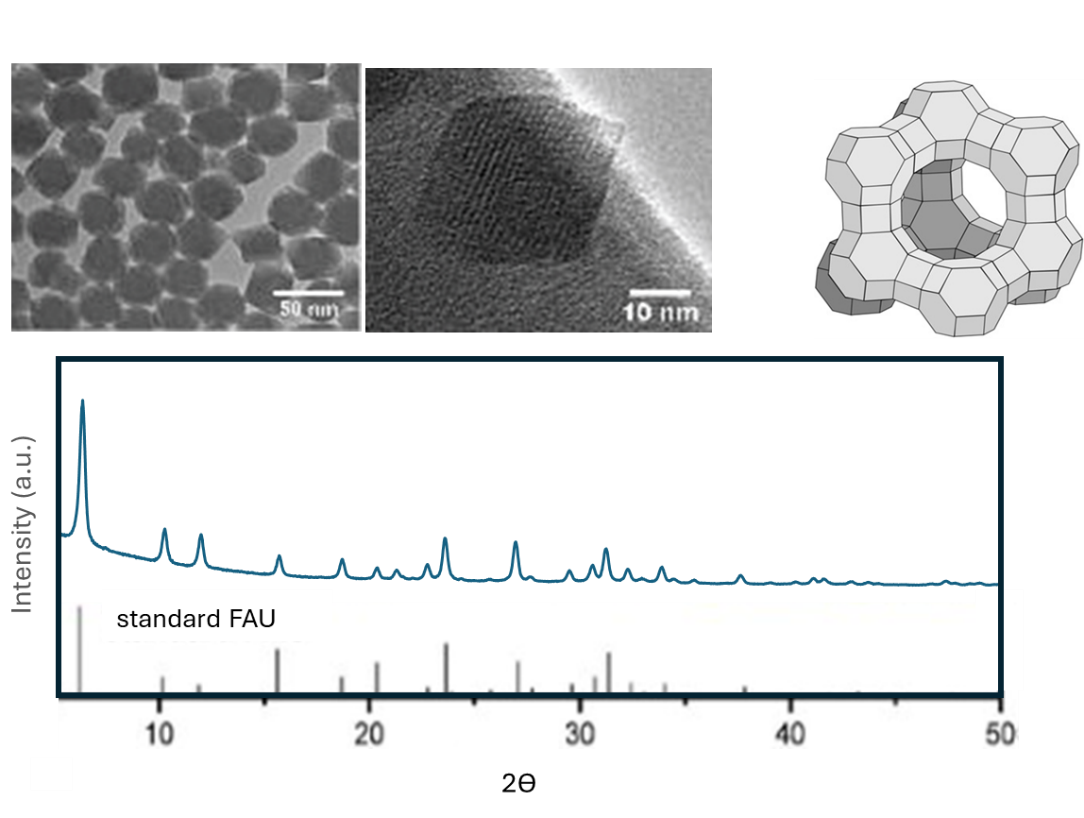


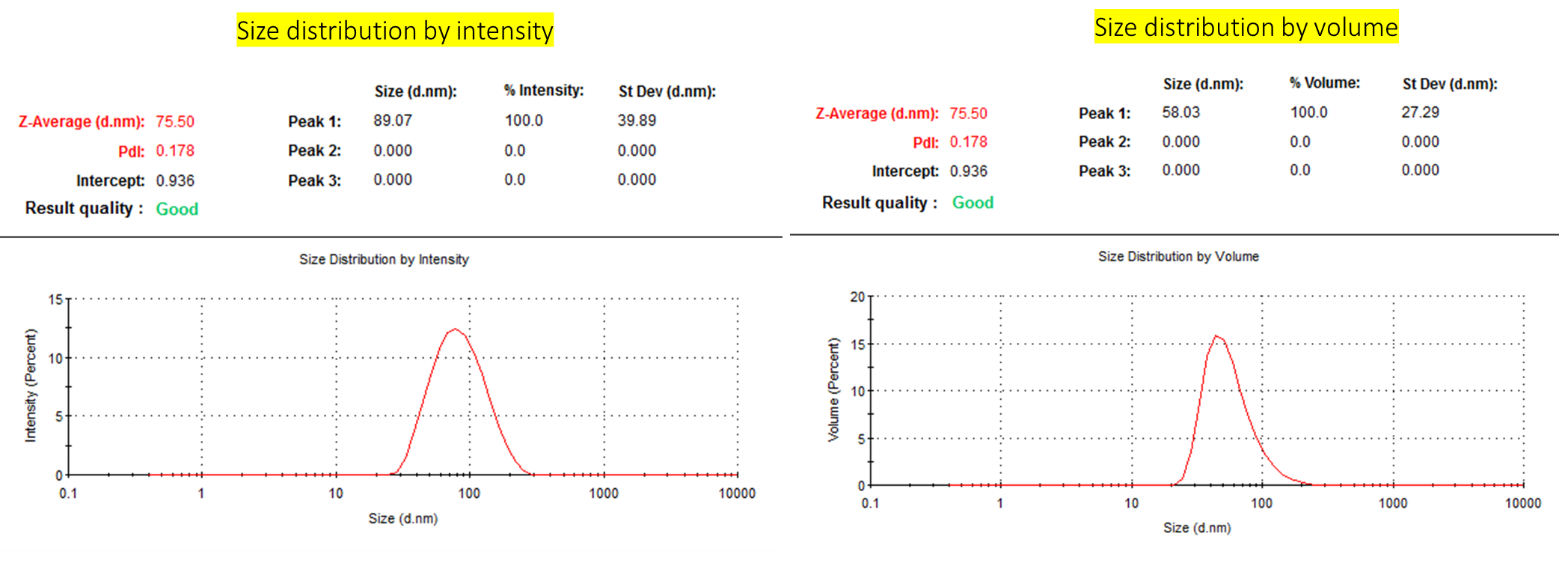


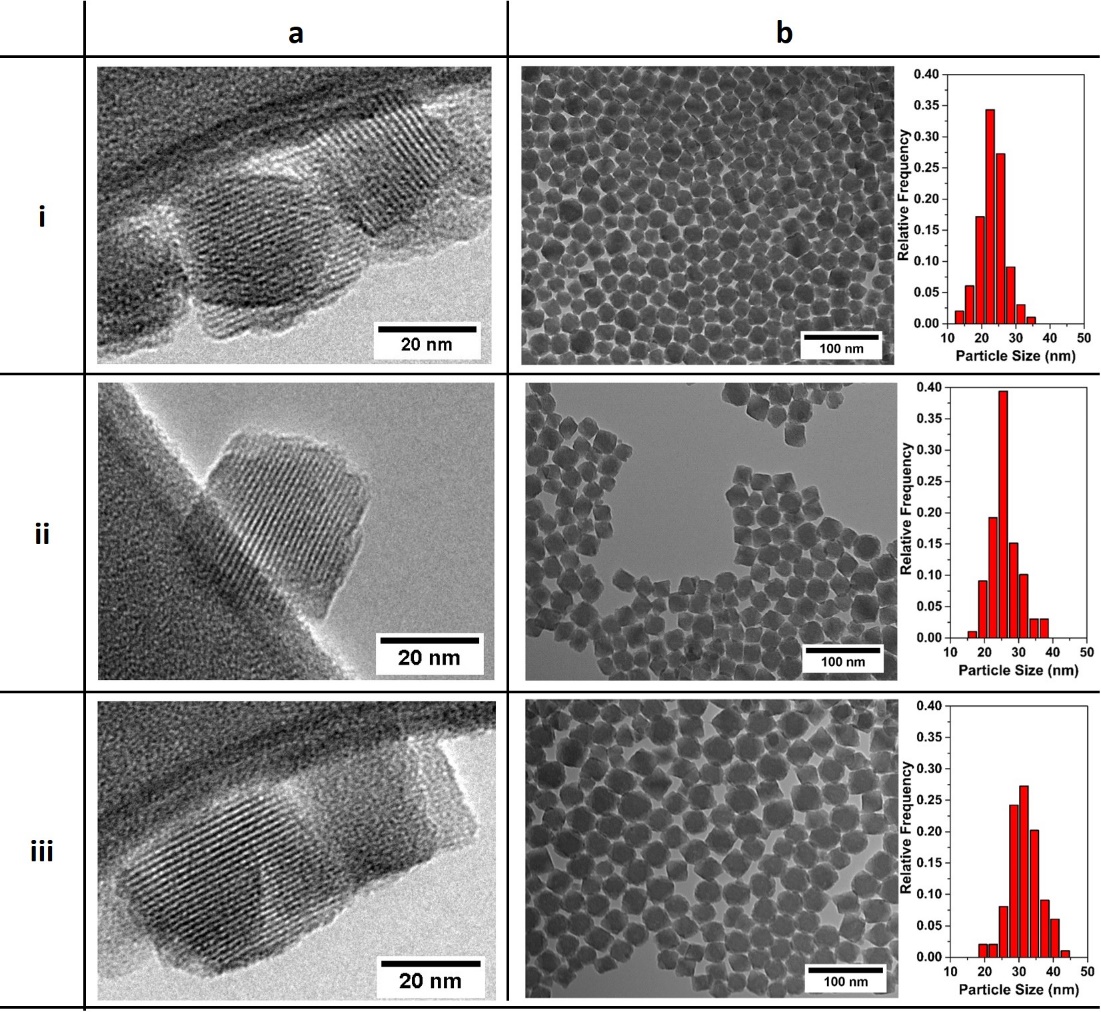


Figure 1S. Powder X-ray diffraction, HRTEM and dynamic light scattering of FAU30 used to make AM30. A schematic of the supercage of FAU30 is shown. Size histogram of FAU30. Volume distribution based on DLS.


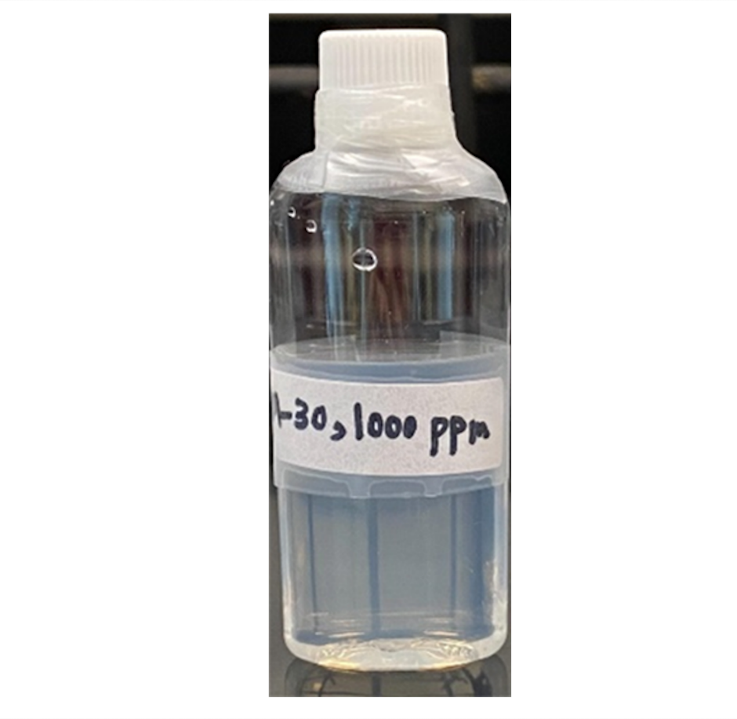


Figure 2S. 1000 ppm solution of AM30 indefinitely stable over time

| **ppm** | 0 | 200 | 100 | 50 | 25 | 12.5 | 6.25 | 3.125 | 1.56 | 0.78 | 0.39 | OD |
| --- | --- | --- | --- | --- | --- | --- | --- | --- | --- | --- | --- | --- |
| **AM30** | 0.715 | 0.046 | 0.363 | 0.689 | 0.812 | 1.026 | 0.849 | 0.867 | 0.803 | 0.801 | 0.812 | 600 |
| **ZnFAU30** | 0.75 | 0.6 | 0.617 | 0.648 | 0.67 | 0.66 | 0.69 | 0.703 | 0.675 | 0.731 | 0.773 | 600 |
| **AgFAU30** | 0.768 | 0.044 | 0.69 | 0.925 | 0.78 | 0.996 | 0.876 | 0.885 | 0.818 | 0.813 | 0.803 | 600 |

| **ppm** | 0 | 200 | 100 | 50 | 25 | 12.5 | 6.25 | 3.125 | 1.56 | 0.78 | 0.39 | OD |
| --- | --- | --- | --- | --- | --- | --- | --- | --- | --- | --- | --- | --- |
| **AM30** | 0.763 | 0.047 | 0.984 | 1.058 | 1.009 | 0.925 | 0.857 | 0.836 | 0.725 | 0.763 | 0.772 | 600 |
| **ZnFAU30** | 0.786 | 0.633 | 0.654 | 0.641 | 0.673 | 0.644 | 0.653 | 0.662 | 0.662 | 0.721 | 0.799 | 600 |
| **AgFAU30** | 0.781 | 0.043 | 0.879 | 1.053 | 0.971 | 0.986 | 0.855 | 0.838 | 0.825 | 0.81 | 0.818 | 600 |

Figure 3S OD600 turbidity readings upon exposures of AM30, ZnFAU30 and AgFAU30 to PAO1 (two experiments). Based on ion-exchange conditions, the concentrations of Zn and Ag in ZnFAU30 and AgFAU30 were similar to AM30.


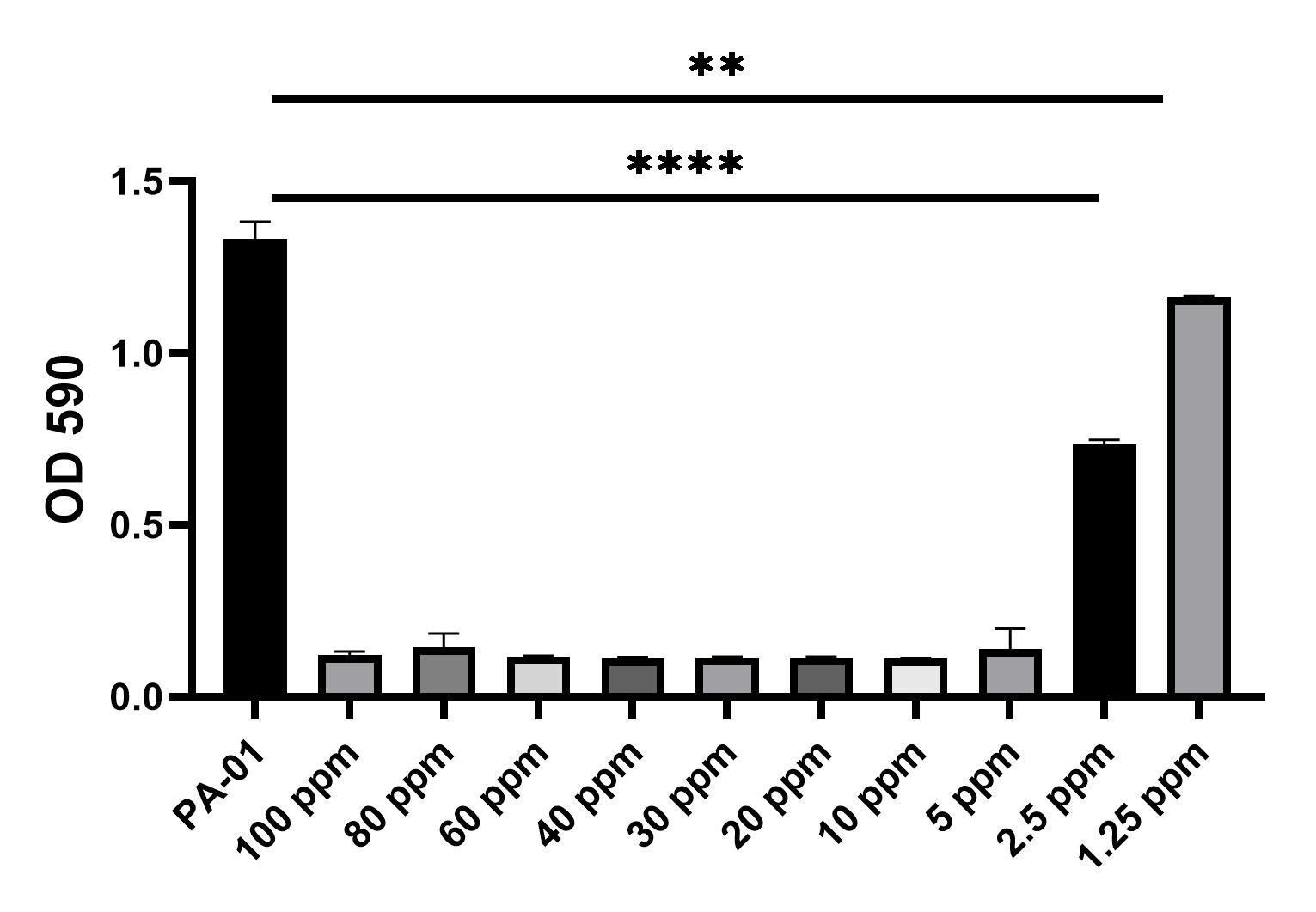


Figure 4S. Biofilm inhibition: Comparison of PAO1 inhibition of biofilm formation in PBS, as measured by the crystal violet assay. AM30 concentrations varying from 1.25-100 ppm, significant inhibition of biofilm formation at concentrations above 5 ppm. Statistical significance was determined using t-test. *p < 0.05, **p < 0.01, ****p < 0.0001


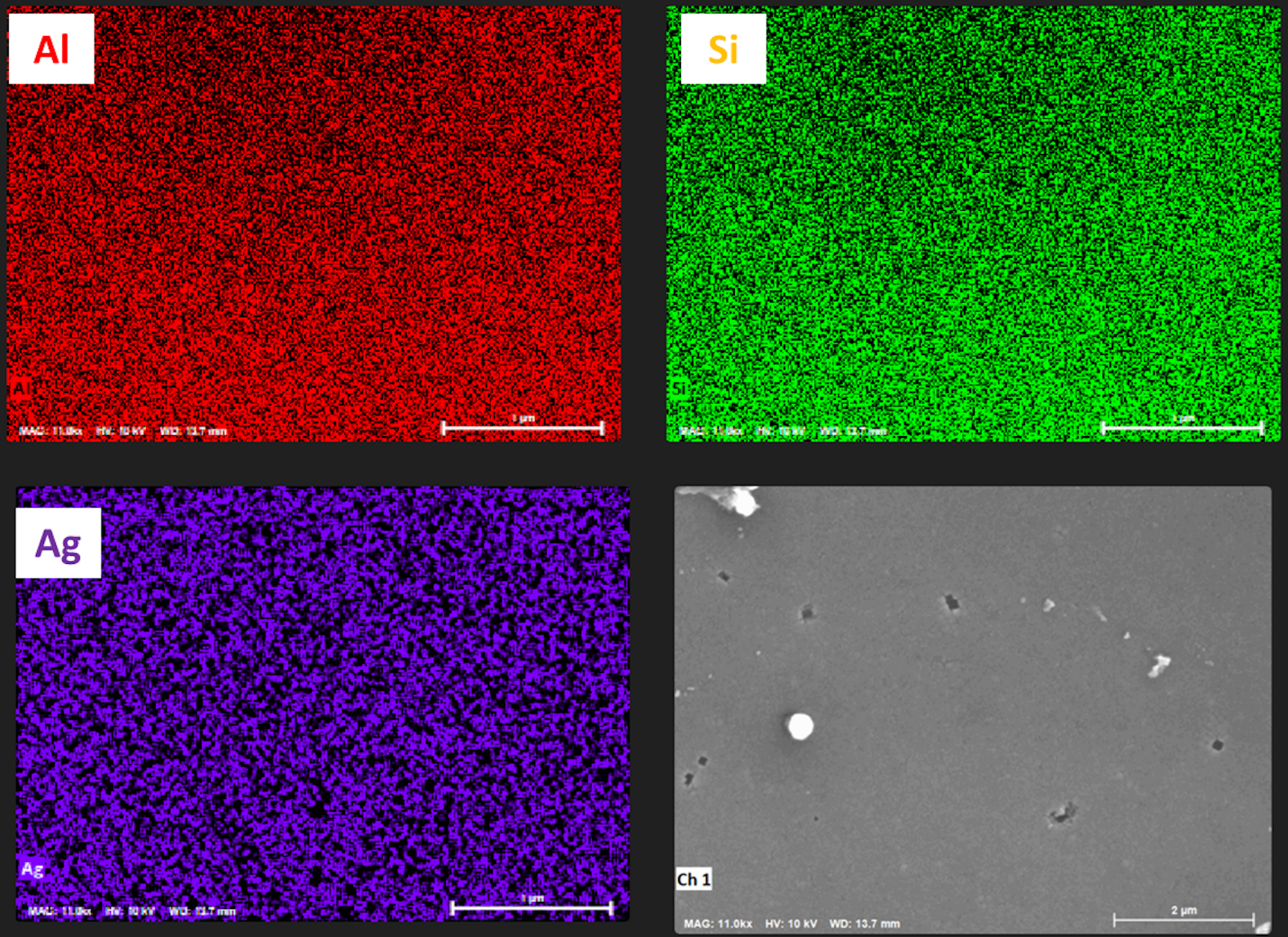


Figure 5S. Elemental map of the surface of ABF-XenoMEM by energy dispersive spectroscopy. Lower right-hand picture is the SEM of ABF-XenoMEM over which the elemental analysis was done.


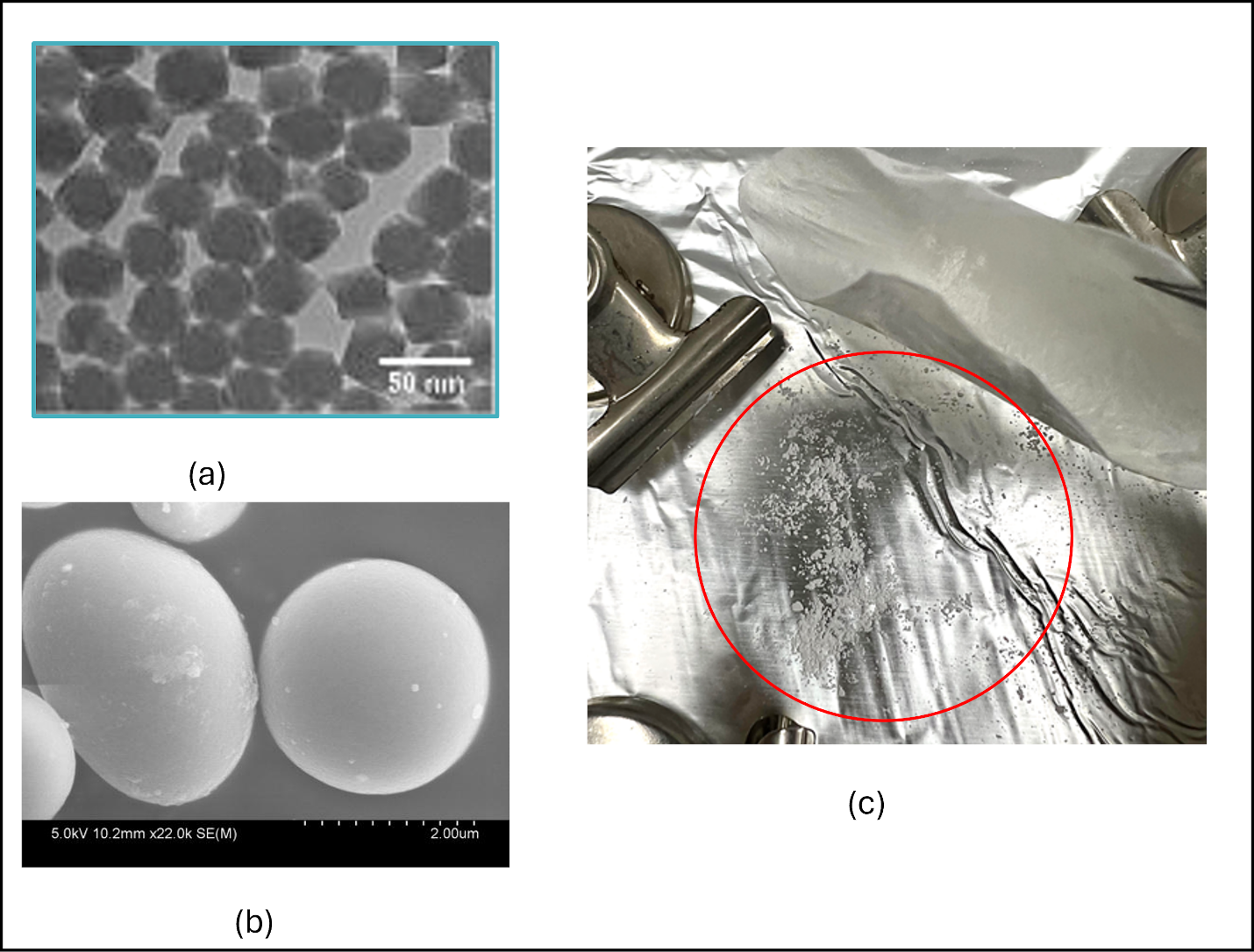


Figure 6S. (a) HRTEM of AM30 (b) SEM of AM30 after spray drying indicating the aggregation and increase in size (c) Photograph of XenoMEM coated with spray dried AM30 gently shaken, leading to the particles falling off from the membrane


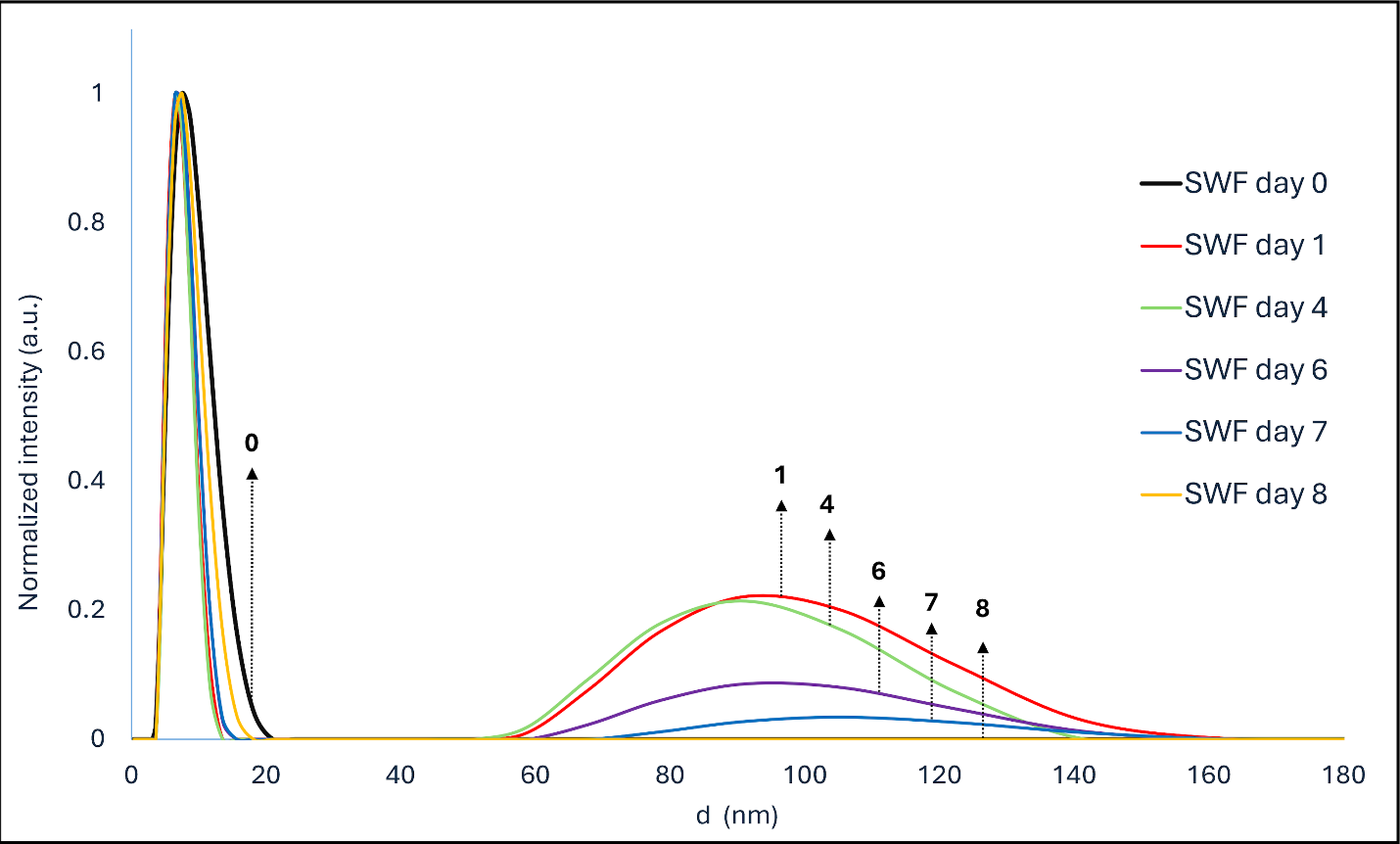


Figure 7S. Dynamic light scattering of SWF (simulated wound fluid) after static exposure to ABF-XenoMEM over time


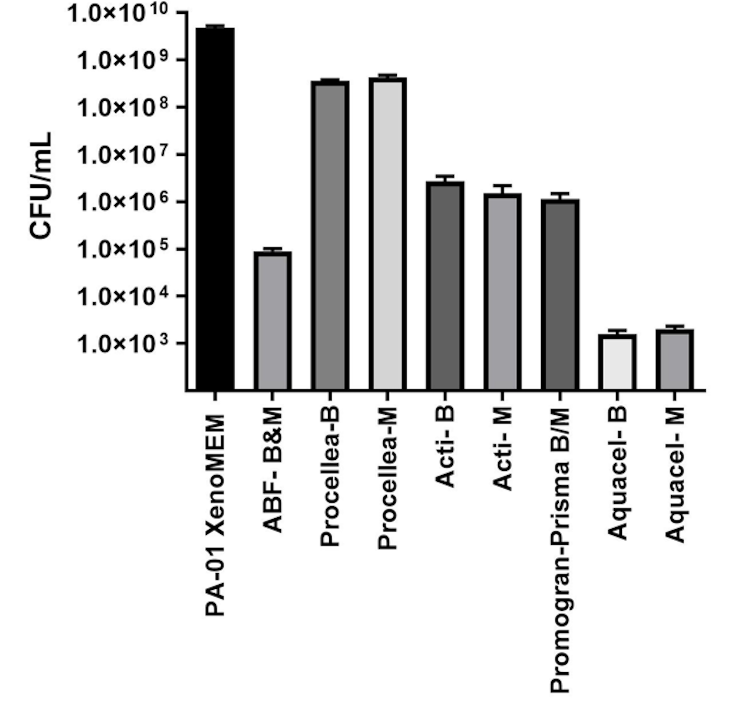


Figure 8S. Comparison of commercial wound dressings and ABF-XenoMEM for biofilm studies. PA01 biofilms were grown on cellulose membrane (M) for 48 h and exposed to ABF-XenoMEM, Procellera^™^, Acticoat^™^ 7, Promogran Prisma^™^ and Aquacel^®^ Ag^+^ Extra^™^ for 24 h (B). The dressing and membrane were separated (except for ABF-XenoMEM and Prisma), and the bacteria on the dressings and membrane were extracted and the bacteria in the extracts were counted (CFU/ml). The data plots the bacteria in the extract observed on B and M separately.


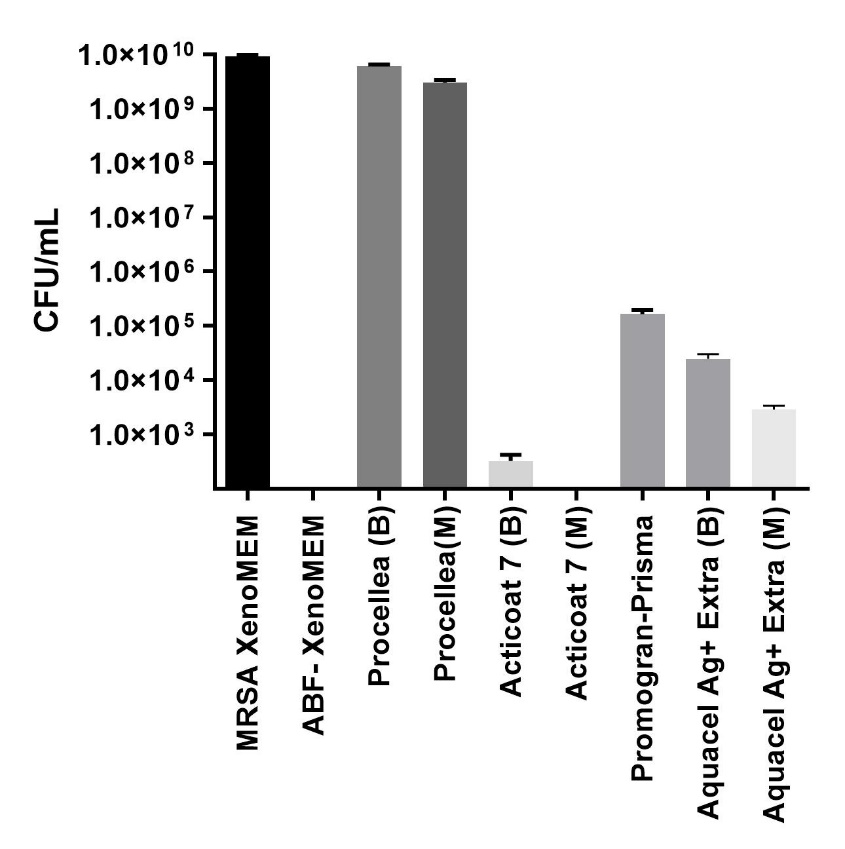


Figure 9S. Comparison of commercial wound dressings and ABF-XenoMEM for biofilm studies. MRSA biofilms were grown on cellulose membrane (M) for 48 h and exposed to ABF-XenoMEM, Procellera^™^, Acticoat^™^ 7, Promogran Prisma^™^ and Aquacel^®^ Ag^+^ Extra^™^ for 24 h (B). The dressing and membrane were separated (except for ABF-XenoMEM and Prisma), and the bacteria on the dressings and membrane were extracted and the bacteria in the extracts were counted (CFU/ml). The data plots the bacteria in the extract observed on B and M separately.
